# Exposure to known and emerging groundwater contaminants significantly alters poultry microbiome and metabolome

**DOI:** 10.1128/aem.02469-25

**Published:** 2026-03-20

**Authors:** Chamia C. Chatman, Elena G. Olson, Steven C. Ricke, Erica L.-W. Majumder

**Affiliations:** 1 Department of Bacteriology, University of Wisconsin-Madison 205263, Madison, Wisconsin, USA; 2 Meat Science and Animal Biologics Discovery Program, Department of Animal and Dairy Sciences, University of Wisconsin-Madison 5228, Madison, Wisconsin, USA; Universidad de los Andes, Bogotá, Colombia

**Keywords:** microplastics, groundwater contaminants, metabolome, microbiome, exposures

## Abstract

The exposome encompasses all lifetime environmental exposures affecting health. Its complexity and high data dimensionality make it challenging to link specific exposure combinations to adverse health outcomes. Establishing relevant exposome criteria is key to addressing current knowledge gaps. This study evaluated contaminant levels in Wisconsin groundwater and their effects on host health. We focused on three co-occurring chemicals that were detected at concentrations exceeding groundwater standards: nitrate, atrazine, and imidacloprid, and the emerging contaminant, microplastics. In this study, broilers were exposed to a low-dose chemical mixture (35,000 ppb nitrate + 1.7 ppb atrazine + 0.58 ppb imidacloprid) and high-dose chemical mixture (100,000 ppb nitrate + 3,000 ppb atrazine + 3,000 ppb imidacloprid) or polyethylene microplastics (PE MPs) for 49 days. Both contaminant types significantly altered the cecal microbiomes as determined by the enrichment of the genera *Fournierella* and *Ruminococcus* and an unclassified *Coriobacteriaceae* genus. Untargeted metabolomics revealed distinct but convergent patterns of metabolic reprogramming across exposures. Chemical mixtures modulated pathways linked to xenobiotic metabolism, pyruvate and thiamine metabolism, and other cofactor-dependent processes, consistent with a shift from oxidative, biosynthetically intensive metabolism toward glycolysis, fermentation, and detoxification. In contrast, PE fibers selectively suppressed oxidative and cofactor/vitamin pathways while perturbing bile acid, sphingolipid, and aromatic compound metabolism, indicating a simplified, maintenance-oriented energy state. Despite these pronounced metabolomic shifts, histopathology revealed no overt intestinal or systemic lesions in any treatment group, highlighting altered microbial activity despite the absence of gross pathological lesions and supporting a silent dysbiosis phenotype.

**IMPORTANCE:**

Environmental contaminants in groundwater are increasingly common, yet their combined effects on animal health remain poorly understood. The current study shows that even low-level exposure to agricultural chemical mixtures and microplastics can alter the gut microbial metabolism in broiler chickens without intestinal damage. These subclinical shifts, characterized by altered energy pathways, cofactor scarcity, and microbial restructuring, highlight a form of silent dysbiosis. Our findings emphasize the need to integrate microbiome- metabolic endpoints into environmental risk assessments to predict earlier, more meaningful, functionally relevant impacts.

## INTRODUCTION

There has been an observed increase in the presence of agricultural chemicals in drinking water (1). However, the health risks associated with chronic exposure remain poorly understood (2). Most risk assessments rely on single-chemical studies, despite evidence that mixtures exert synergistic or compensatory effects on host physiology and microbial function. Xenobiotics, including pesticides and emerging contaminants such as microplastics (MPs), enter livestock and humans primarily through ingestion (3–6). However, inhalation and dermal contact are also routes of exposure for MPs (5).

The gut microbiota plays a central role in biotransforming ingested xenobiotics into metabolites that influence mucosal integrity, immune function, and energy metabolism (3). Although xenobiotic phase I/II metabolism occurs in the small intestine and liver (7, 8), agricultural chemicals and microplastics have been demonstrated to perturb the gut microbiome responsible for these transformations. Previous studies demonstrate species- and chemical-dependent sensitivity. In amphibians, chronic levels of atrazine resulted in disruption of microbial community and metabolic pathways (9, 10). Imidacloprid exhibits different responses in honeybees and zebrafish. While zebrafish elicited a significant impact on gut microbial composition (11), honeybees did not exhibit signs of perturbed gut microbiomes but increased in mortality (12). These inconsistencies highlight the need for controlled cross-species *in vivo* studies that evaluate microbiome responses to complex chemical mixtures. Microplastics pose an additional emerging concern. Primary MPs, manufactured particles, and secondary MPs, environmentally degraded plastics, have been detected in diverse human tissue including placenta and testes (13–18), and livestock (19), exacerbating public health concerns. Variability of biological impact depends on the polymer type, shape, and size (4–7). Livestock, including poultry, may be exposed via ingestion (20); however, a few studies have examined MP effects on gut microbial function in food-producing animals.

Here, we evaluated the effects of known contaminants, a mixture of nitrate, imidacloprid, and atrazine, and the emerging contaminant, polyethylene fiber microplastics (PE fiber MPs), using broiler chickens as an *in vivo* model system. Broilers represent a highly relevant system due to widespread consumption (21) and high sensitivity to environmental factors affecting microbial ecology and growth performance. The ceca are the primary site for microbial activity in the poultry gut (22) and thus an ideal environment to explore xenobiotic metabolism via microbiome-metabolome responses. By integrating 16S rRNA sequencing and untargeted metabolomics (23), we assessed how these contaminants modulate microbial structure and functional metabolic pathways. Chemical mixture and concentrations were selected due to their frequency of detection and concentrations exceeding enforcement standards in the 2021 Department of Agriculture, Trade, and Consumer Protection (DATCP) Targeted Sampling Report (24). In addition, PE MPs were incorporated as the emerging contaminant of interest based on our previous research, which indicated that polyethylene (PE) fiber MPs alter the taxonomic composition of cecal microbiomes within *in vitro* cecal mesocosms (25). We hypothesized that exposure to both contaminant types would disrupt cecal microbial structure and function, with distinct mechanisms of metabolic compensation subsequently impacting cecal microbial activity via modulation of key metabolic pathways *in vivo*.

In the current study, we conducted a 49-day *in vivo* exposure trial in broiler chickens to evaluate how environmentally relevant groundwater contaminants and PE fiber MPs influence gut microbial structure and function. Using a controlled floor-pen design, we exposed birds to two concentrations of a ternary mixture (nitrate-atrazine-imidacloprid) or PE fibers incorporated into the diet. We then integrated 16S rRNA gene sequencing with untargeted metabolomics to assess how these contaminants reshape cecal community composition, energy metabolism, and functional pathway activity. This multi-omics approach allowed us to identify early, subclinical indicators of contaminant-driven metabolic stress that are not captured by conventional pathology.

## MATERIALS AND METHODS

### Animal husbandry and experimental design

Aviagen Ross 308 Broiler chickens (mixed sex) were purchased from Welp Hatchery in Bancroft, Iowa. Broilers were housed at the Poultry Research Facility at the University of Wisconsin-Madison. Birds were randomly assigned to a treatment group and immediately placed into the designated floor pen. The broilers (*N* = 100) were allowed to acclimate to the facility for 7 days. Water and commercial poultry diet (Agrimaster 21% Meat Producer Poultry Feed Crumbles; Blain #334839, Mfr #3931) were fed *ad libitum* (Tables S1 and S2). The Agrimaster diet is designed to be fed to poultry during the starter, grower, and finisher phases of broiler production.

### Preparation of polyethylene microplastics

Following the methods by Cole (26), low-density polyethylene (LDPE) fibers (GoodFellow, LS554234) were cut to 50 µm using a cryotome (Leica Biosystem 1950 cryotome). Polyethylene fiber sizes were subsequently verified using scanning electron microscopy (Zeiss GeminiSEM 450) (27). Polyethylene fibers (250 mg/kg poultry basal diet) were then added to the poultry basal diet weekly to account for changes in feed consumption during the starter, grower, and finisher phases of broiler production. For this study, the microplastic concentration was based on the median concentration of microplastics detected in a terrestrial environment in the USA (28). A similar concentration was also used for an *in vivo* MP toxicity study (29).

### Selection and preparation of groundwater contaminant chemicals

We selected a mixture of nitrate, atrazine, and imidacloprid based on their frequency of detection and concentrations exceeding enforcement standards in the 2021 DATCP Targeted Sampling Report (24). Chemicals used in this study were atrazine (ASTATACH, Lot: P151-04287), imidacloprid (Thermo Scientific, Lot: A0450203), and nitrate (AccuStandard, Lot: 222105041). Atrazine was first diluted in dimethyl sulfoxide (DMSO), and then, the final dilutions were done in autoclaved ultrapure water. Nitrate and imidacloprid dilutions were done in autoclaved ultrapure water.

In our previous study, nitrate, atrazine, and imidacloprid were selected due to their frequency of detection in groundwater wells and chemical concentrations for each exceeding either an enforcement standard or preventative action limit (30). One of the three treatment groups represented chemical concentrations analogous to those detected in the 2021 DATCP Targeted Sampling Report. We then used *in silico* and *in vitro* methodologies to further evaluate the DATCP report and elucidate if potential chemical-biological interactions would be present between the chemical mixtures and our selected models, Caco-2 cells, and poultry cecal microbiota. We concluded that the poultry cecal microbiota exposed to the ternary mixtures had a decline in growth rate (30). However, Caco-2 cells exposed to various two-chemical combinations of nitrate, atrazine, and imidacloprid did elicit a significant decrease in cell viability, but not the ternary mixtures (30).

For this *in vivo* trial, the low-dose chemical mixture represents chemical concentrations like those detected in the 2021 DATCP Targeted Sampling Report (24) (Table 1). The high-dose mixture was based on preliminary exposure studies where it was observed that two-chemical combinations of each chemical (i.e., nitrate + atrazine, nitrate + imidacloprid, and atrazine + imidacloprid) at 3,000 ppb resulted in growth inhibition to poultry cecal organisms *in vitro* (30). To elucidate if 3,000 ppb of each chemical would result in similar perturbation to the poultry cecal microbiome and metabolome *in vivo,* this concentration was selected for atrazine and imidacloprid only due to the environmental concentration of nitrate exceeding this 3,000 ppb. Furthermore, atrazine and imidacloprid concentrations used for the high-dose chemical mixture exceeded the preventative action limits for each chemical (31). The concentration of nitrate was set higher than detected levels and greater than the preventative action limit for nitrate (31). Although the high-dose treatment exceeded current preventive action and enforcement standards, it was intentionally kept below levels previously observed in laboratory experiments (32, 33).

**TABLE 1:** Composition of agricultural chemical mixtures used in this study

	Nitrate concentration (ppb)	Atrazine concentration (ppb)	Imidacloprid concentration (ppb)
Low dose	35,000	1.7	0.58
High dose	100,000	3,000	3,000

### Forty-nine-day MP exposure study in broilers

Following the acclimation period, broilers were randomly assigned to one of four treatment groups. The treatment groups are as follows: untreated control group (*n* = 17), polyethylene fiber treatment group (+PE Fiber; *n* = 20), low-dose mixture (*n* = 22), and high-dose mixture (*n* = 19). The treated feed was prepared weekly by pouring each treatment into a designated feed bin while gently mixing the feed. On day 49, all broiler chickens were humanely euthanized with carbon dioxide asphyxiation. Ceca from each bird was separated at the ileal-cecal junction with sterile scalpel blades and disposable forceps. The cecal digesta was divided into two aliquots and flash-frozen with liquid nitrogen.

### Evaluation of growth parameters

Individual bird weights were recorded on days 0, 7, 14, 28, 35, 42, and 49. Broilers’ individual weights were subsequently averaged to account for total body weight gain per treatment (accounting for mortalities). Body weight gain was defined as the ratio of total body weight gain and the number of days on trial. To monitor feed consumption, the feeders were also weighed per pen on days 0, 7, 14, 28, 35, 42, and 49. Mortalities were not included when calculating feed conversion ratios due to final carcass weights not being recorded. Feed conversion ratio (FCR) is the ratio of the total feed intake (per pen) and total weight gain (per pen). Feed intake (per pen) is defined here as the difference in weight of feed bins pre- and 1-week post-feeding for each treatment group. For all statistical analyses, PE fiber was only compared to control (i.e., control vs. PE fiber), and chemical mixture treatment groups were compared to each other and the control (i.e., control vs. low dose, control vs. high dose, and low dose vs. high dose). One-way analysis of variance (ANOVA) statistical analysis was performed on BWG parameters in GraphPad Prism (version 10.4.0) (Brown-Forsythe and Welch ANOVA test; *P*-value ≤ 0.05). To further compare mean BWG across groups, a Dunnett’s multiple comparison test was conducted (*P*-value ≤ 0.05). Descriptive statistics were performed in GraphPad Prism (version 10.4.0) to compare the mean feed conversion ratio across treatment groups. Statistical analysis was then performed for feed intake (FI) data in R (version 4.4.0) (Linear Mixed Effects Model; *P*-value ≤ 0.05).

### Histological evaluation

All the tissues were evaluated blindly and scored by the American College of Veterinary Pathologists (ACVP) board-certified Veterinary Pathologist at the University of Wisconsin-Madison’s Comparative Pathology Laboratory using standard methods (34–36). In brief, tissues were harvested from the same anatomical location from all the animals (*N* = 78). Tissues evaluated included liver, spleen, kidney, duodenum, pancreas, ceca, and colon. All tissues were collected and fixed in 10% buffered formalin for 24 h and transferred to 70% ethyl alcohol until processing. Formalin-fixed tissue sections were processed by a Sakura Tissue-TEK VIP and paraffin-embedded before sectioning onto a glass slide. Tissue sections (5 µm) were observed using an optical microscope (Olympus Bx 43, Software CellSens Standard). The table scoring system used a four-point scale (0, none; 1, minimal; 2, mild; 3, moderate; and 4, severe changes). Tissue sections were evaluated for the following perturbations. Livers were assessed for vascular changes, including peliosis hepatis-like lesions, apoptosis, vacuolar changes, and hepatic lipidosis; spleens for lymphoid depletion; kidneys for renal tubular hydropic/vacuolar degeneration and formation of vacuoles; small and large intestines for changes of enteric and enteric epithelial architecture; and pancreas for exocrine and endocrine pancreas.

### Library preparation and 16S rRNA gene amplicon community sequencing

DNA extraction was performed using the standard protocol for the DNeasy Blood and Tissue kit (Qiagen, Cat 69506). Cecal samples from each bird were collected and stored in −80°C until genomic DNA extractions were performed (*N* = 78; control = 17, +PE fiber = 20, low-dose chemical mixture = 22, and high-dose chemical mixture = 19). A 0.5 mL aliquot from each bird was used for gDNA extractions. Samples were centrifuged for 5 min at 14,000 × *g,* and the supernatant was subsequently discarded. Total genomic DNA quality and concentration were verified using an Infinite 200Pro spectrophotometer (Tecan Nano Quant Plate). The DNA extracts were diluted to 10 ng/µL in Buffer AE and stored at −80°C until further analysis.

To initiate amplicon library preparation for microbiome sequencing, DNA extracts were PCR-amplified with primers targeting the V4 region of the 16S rRNA gene. The primers were dual-indexed primers designed using high-fidelity polymerase, PhiX, according to the protocol by Kozich et al. (37). Gel electrophoresis was performed to verify amplified PCR products. The amplified PCR products were then normalized to 20 µL using a SequalPrep Normalization kit (Life Technologies, Carlsbad, CA, USA). Aliquots of 5 µL from each normalized sample were subsequently pooled into the final library. Final concentrations were verified using a KAPA library quantification kit (Kapa Biosystems, Wilmington, MA, USA) and a Qubit 2.0 Fluorometer (Invitrogen, Waltham, MA, USA). Next, the final library was diluted to 20 nM with HT1 buffer and PhiX control v3 (20%, vol/vol), and 600 µL was loaded onto a MiSeq v2 (500 cycles) reagent cartridge (Illumina, San Diego, CA, USA) to begin the sequencing run.

### Microbiome community analyses

The raw Illumina amplicon sequence data were uploaded to the BaseSpace website (Illumina, San Diego, CA, USA) to assess sequence run quality and completion. Quantitative Insights Into Microbial Ecology (QIIME) 2 (version 2024.5) (38) was utilized to perform microbiome bioinformatics. The demultiplexed data were downloaded from the Illumina Basespace website and uploaded to QIIME2 using Casava 1.8 paired-end demultiplexed format (via QIIME import tools). The DADA2 (39) data (via q2-dada2) were subsequently quality-filtered using the chimera consensus pipeline. Microbiome samples used for comparing control versus +PE Fiber treatment groups were filtered to a sampling depth of 3,002, which retained 84,056 features (16.70%) in 77.78% of samples (*N* = 28). Microbiome samples comparing control, low-dose mixture, and high-dose mixture were filtered to a sampling depth of 1,143, which retained 49,149 features (8.46%) in 74.14% of samples (*N* = 43). Alpha rarefaction plots were used to confirm the sampling depth. The phylogenetic tree (via q2-phylogeny) was generated by aligning the amplicon sequence variants with mafft (40) (via q2-alignment) and fastree2 (41). Taxonomy was subsequently assigned to the ASVs in the feature table using (via q2-classifier sklearn) (confidence limit of 97%) (42). The classifier was trained using the SILVA 138 99% operational taxonomic unit reference sequences (43).

To identify differentially abundant features across treatment groups, Analysis of Compositions of Microbiomes with Bias Correction (ANCOM-BC) was used. ANCOM-BC is a linear regression model that corrects for bias by accounting for differential sampling fractions across samples (44). A pairwise comparison using Kruskal-Wallis was conducted for the following α-diversity metrics: Shannon’s Diversity Index, Observed Features, Faith’s Phylogenetic Diversity, and Pielou’s Evenness (*P*-value ≤ 0.05; Q-value ≤0.05) (45). Main effects and interactions of variables were assessed using analysis of variance (ANOVA) for α-diversity metrics. Treatment effects based on β-diversity metrics (i.e., Jaccard distance, Bray-Curtis distance, unweighted UniFrac distance, and weighted UniFrac distance) (via q2-composition) were assessed using non-parametric multivariate analysis of variance (PERMANOVA) (*P*-value ≤ 0.05; Q-value ≤0.05) (46, 47). For both α-diversity and β-diversity metrics, PE fiber was only compared to control (i.e., control vs. PE Fiber) and chemical mixture treatment groups were compared to each other and the control (i.e., control vs. low dose, control vs. high dose, and low dose vs. high dose).

### Metabolite extraction

Prior to metabolite extraction, ~1 g of cecal digesta was diluted in 2 mL of autoclaved water. An aliquot (0.3 mL) of the diluted cecal digesta was used to determine protein concentration by a Bradford assay (48). Metabolite extraction was performed using 0.5 mL of diluted cecal digesta from each broiler. Cells were lysed by three freeze-thaw and sonication cycles, which consisted of thawing at room temperature for 10 min, followed by a 30-s sonication on ice in a water bath sonicator (Branson 2800). A 1 mL aliquot of 2:2:1 (vol/vol) mixture of ≥99.9% purity acetonitrile (Fisher Scientific; Cat.A998-4) ≥99.8% purity methanol (Fisher Scientific; Cat. A412-4), and water was added to cell extracts and sonicated for 30 s and stored at −20°C overnight to allow cellular debris and protein to precipitate. The next day, samples were centrifuged for 15 min at 20,784 × *g* at 4°C. The supernatant was transferred to new microcentrifuge tubes and dried for 6 h on a SpeedVac Concentrator (Thermo Scientific Savant DNA 120). Dried samples were then reconstituted in acetonitrile:water (1:1 vol/vol) solvent mixture based on the normalized protein concentration of all samples, where the highest protein concentration sample had 100 μL of resuspension volume. Samples were vortexed for 30 s, sonicated on ice for 10 min, and centrifuged for 15 min at 13,000 rpm at 4°C to remove any residual debris. The metabolite extracts were transferred into 0.3 mL high-performance liquid chromatography (HPLC) autosampler vials with inserts (VWR International LLC, Cat. 9532S-1CP-RS) and stored in −80°C until analysis.

### Untargeted metabolomics

Using a protocol by Chatman et al. (2024) (49), untargeted metabolomics was conducted. Metabolite extracts were analyzed with ultra-high-performance liquid orbitrap chromatography mass spectrometry (UHPLC-MS) (Thermo Scientific Orbitrap Exploris 240 mass spectrometer). A Kinetex Core-Shell 100 Å column C-18 column (1 × 150 mm, 1.7 μm, Phenomenex) was used for the separation of metabolites. Sample injection volume was 3 μL at a flow rate of 0.250 mL/min. Mobile phase A was composed of water with 0.1% formic acid, and mobile phase B was 0.1% formic acid in acetonitrile. The gradient began with 5% B from 0 to 3 min, then an increase and hold to 95% B until 18 min, followed by a decrease to 5% B at 18.50 min and held at 5% B until 22 min. Data-dependent acquisition was used for the tandem MS workflow in positive ion mode. Twenty-eight of the extracts were run successfully before a UPLC malfunction; however, due to our practice of randomizing samples in the run order and including QA and QC samples, there were sufficient replicates from each group to proceed with metabolomics data analysis.

### Untargeted metabolomics data analysis

MetaboAnalyst 6.0 was used for statistical analysis and functional analysis of the MS1 data as described in Pang et al. (2022) (50). The raw files were converted to mzML files and centroided (MS-1, Orbitrap, and positive mode) using ProteoWizard version 3.0 (51). Univariate analyses were performed to investigate statistical differences in metabolite features between treatment groups. Principal component analysis provided insight into similarities and differences among the treatment group. Analysis of variance analysis (ANOVA) was conducted to investigate potential differences in metabolite features between each treatment group (Tukey’s post-hoc test; *P*≤ 0.05). Hierarchical clustering was also conducted to evaluate the top 25 features across treatment groups (distance measure: Euclidean, clustering method: Ward; *P*-value = 0.05). Functional analysis (*P*-value = 0.05, KEGG *Gallus gallus* library) was conducted to assess pathway-level changes to the cecal metabolome. Feature annotation of metabolites was first carried out using MetaboAnalyst 6.0, which matches MS1 data compounds in Human Metabolome Database. Functional analysis was subsequently conducted to assess pathway-level changes to the cecal metabolome. Compound Discoverer (Version 3.3, Thermo Fisher Scientific) was used for statistical, identification, and functional analyses of raw MS/MS data. Raw MS/MS files in Compound Discoverer were compared against mzCloud, Metabolika, and ChemSpider databases.

## RESULTS

### Broiler chicken growth performance and health outcomes after exposures: agricultural groundwater contaminant and microplastic exposure altered growth metrics but not pathology

To assess the impact of PE fiber MPs on broiler growth performance, average body weight gain (BWG), feed intake (FI), and feed conversion ratio (FCR) were calculated per pen (Table 2). Overall, there was no significant change to BWG from D0 to D49 when comparing the control and +PE fiber MPs treatment group (Table 2). However, we observed that BWG for the +PE fiber treatment group was significantly lower at D28 to 35 compared to the control (*P*-value ≤ 0.05) (Table 2). Analysis of the mean FCR over the treatment period (49 days) indicated that the +PE Fiber treatment group did have a lower mean FCR (Table 2). Analysis of FI was also performed to determine if exposure to agricultural contaminants negatively impacted broilers’ feed consumption. The +PE fiber treatment group exhibited significantly different intake patterns from control birds (Fig. S1; *P* < 0.001). Although FI patterns appear similar, the presence of PE Fiber in the poultry diet increased FI at later growth stages, particularly after D28 (Fig. S1; *P* < 0.001).

**TABLE 2:** Summary of body weight gain (BWG) and feed conversion ratio (FCR) results comparing control and +PE fiber treatment groups^*a*^

BWG (kg/bird/week)	Control mean (*n* = 17)	+PE fiber mean (*N* = 20)	*P*-value^*b*^	SEM
D0–7	0.00	0.00	0.16	0.00
D7–14	0.01	0.01	0.48	0.00
D14–28	0.01	0.01	0.71	0.00
D28–35	**0.06**	**0.01**	**<0.00**	**0.00**
D35–42	0.12	0.02	0.48	0.00
D42–49	0.02	0.02	0.52	0.00
D0–49	0.07	0.07	0.26	0.00

FCR (kg/pen)	Mean	SEM
Control (n = 17)	2.34	0.03
+PE fiber (n = 20)	2	0.14

^*a*^ Bolded values indicate significance (Brown-Forsythe ANOVA test; *P*-value ≤ 0.05).

^*b*^ The *P*-value is computed from the F* ratio and is analogous to the F ratio commonly used for ANOVA.

We also observed significant differences in BWG for the ternary mixture treatment groups from D7 to D14, D14 to D28, D28 to D35, and D42 to D49 (Table 3). Analysis of the mean FCR of control, low-dose, and high-dose mixture treatment groups over the treatment period (49 days) indicated that the low-dose mixture and high-dose mixture treatment groups have a lower mean FCR compared to the control group (Table 3). FI was also assessed to evaluate feed consumption over the treatment period. FI increased significantly over the treatment period for control, low-dose, and high-dose groups (Fig. S2). However, at D28, FI for low-dose and high-dose treatment groups begins to significantly differ from the control group (Fig. S1; *P* < 0.001).

**TABLE 3:** Summary of body weight gain and feed conversion ratio results comparing control, low-dose mixture, and high-dose mixture treatment groups^*a*^

BWG (kg/bird)	Control mean (*N* = 17)	Low-dose mixture mean (*N* = 22)	High-dose mixture mean (*N* = 19)	*P*-value^*b*^	F*ratio			dFn, dFd^*c*^
D0–7	0.004	0.003	0.003	0.87	0.14			2
D7–14	0.009	0.007	0.012	**0.00**	7.39			2
D14–28	0.004	0.013	0.013	**<0.00**	162			2
D28–35	0.069	0.011	0.011	**<0.00**	313			2
D35–42	0.019	0.018	0.017	0.42	0.87			2
D42–49	0.017	0.021	0.018	**0.02**	4.15			2
D0–49	0.067	0.071	0.018	**<0.00**	365			2

FCR (kg/pen)	Mean	SEM
Control (*n* = 17)	2.34	0.03
Low-dose mixture (*n* = 22)	1.87	0.26
High-dose mixture (*n* = 19)	1.90	0.24

^*a*^ Bolded values indicate significance (Brown-Forsythe ANOVA test; *P*-value ≤ 0.05).

^*b*^ The *P*-value is computed from the F* ratio and is analogous to the F ratio commonly used for ANOVA.

^*c*^ Degrees of freedom for the numerator (dFn) and denominator (Dfd) used for calculating the F*ratio.

Histological evaluation was then conducted to evaluate if pathological damage occurred following exposure to the +PE fiber, low-dose, and high-dose treatment groups. There were no overt signs of inflammation, lesions, abnormal cellular development, or other significant changes to the intestinal tract in any group (Fig. S3 and S4). In addition, mortality rates over the trial did not appear to be correlated to treatment due to several of the control group mortalities being attributed to wing band injuries early in the study period and heat stress at later stages of the trial. However, the high-dose group did have four mortalities reported from D7 to D14, unlike the low-dose group, which had zero from D7 to D42 (Fig. S5). Together, these findings indicate that while neither PE fiber microplastics nor chemical mixtures caused overt pathological damage, both exposures altered bird physiology sufficiently to warrant investigation into microbial and metabolic responses. Therefore, subsequent analyses focused on how these treatments modulated the cecal microbiome and its associated metabolome.

### Effects of agricultural groundwater contaminant chemical mixtures on cecal microbiome and metabolome

#### High-dose mixtures restructured microbial diversity and community composition

**Fig 1:**
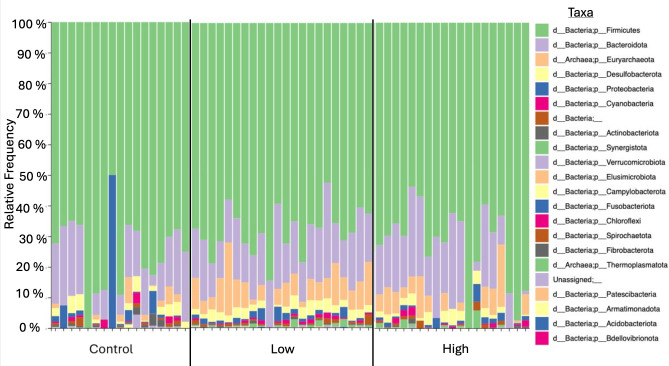
Taxon bar plot showing the relative frequency of phyla by treatment group. Treatment groups are separated by a solid black line.

In this study, 16s rRNA amplicon sequencing was utilized to evaluate changes in the cecal microbial composition following exposure to low and high concentrations of a ternary mixture of nitrate, atrazine, and imidacloprid. Overall, the cecal microbiomes of each treatment group exhibited an abundance of *Firmicutes* and *Bacteroidota* (Fig. 1). This corresponds with our previously sequenced poultry microbiomes (49, 52, 53). In addition, broilers in the low-dose mixture and high-dose mixture treatment groups had a higher relative frequency of *Euryarchaeota* (Fig. 1).

To assess treatment effects on microbial composition, ANOVA for α-diversity metrics was evaluated. The results indicated that the main effect, “treatment” (low-dose and high-dose ternary mixtures), significantly impacted microbial diversity and richness (Table S3) (*P*-value ≤ 0.05). Further evaluation of treatment effects on microbial composition was conducted using Kruskal-Wallis pairwise comparisons. Comparison of the control and high-dose mixture treatment groups detected significance for α-diversity metrics, Faith’s phylogenetic diversity, and observed features (Table S3) (*P*-value ≤ 0.05; Q-value ≤ 0.05). However, Kruskal-Wallis pairwise results for a-diversity metrics were not significant for comparisons of the control group and low-dose mixture treatment groups (Table S6) (*P*-value ≥ 0.05; Q-value ≥0.05). Pairwise comparison of the high-dose mixture treatment group to the low-dose mixture treatment group did yield significance for Shannon’s diversity index, Faith’s phylogenetic diversity, and observed features (Table S4) (*P*-value ≤ 0.05; Q-value ≤ 0.05).

We then evaluated treatment effects as related to β-diversity metrics. First, PERMANOVA was used for β-diversity metrics to assess significant differences in taxonomic composition between groups. The main effect “treatment” was determined to significantly impact cecal microbial composition based on β-diversity metrics (Table S5) (*P*-value ≤ 0.05; Q-value ≤ 0.05). Significance was detected for all comparisons (Table S5). The results were subsequently visualized using principal coordinate analysis in Emperor. PCoA plots provided a visual indication of similarities and dissimilarities among the treatment groups. It was determined that the low- and high-dose mixture treatment groups clustered more closely together, indicating similarity in microbial composition (Fig. S6).

**Fig 2:**
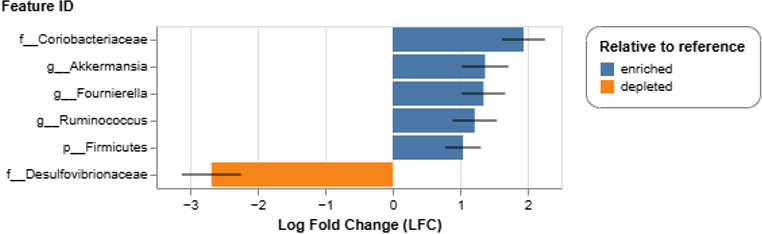
Differential abundance of genera in the low-dose and high-dose groups relative to the control group as determined by ANCOM-BC. Unclassified genera were annotated to the family level.

Next, differentially abundant taxa were determined using ANCOM-BC. Differential abundance analysis as related to the control group revealed that the genera *Akkermansia*, *Fournierella*, *Ruminococcus,* and an unclassified genus in the family *Coriobacteriaceae* were enriched in the low-dose and high-dose mixture treatment groups (Fig. 2). One taxon was depleted in the control group, which was identified as an unclassified genus in the family *Desulfovibrionaceae*. These data further confirmed the dissimilarity between the control and ternary mixture treatment groups. Given that chemical mixtures induced marked compositional shifts in the cecal microbiome, we next evaluated whether these taxonomic changes corresponded to alterations in metabolic potential and functional activity.

#### Chemical mixtures produced distinct cecal metabolic signatures

**Fig 3:**
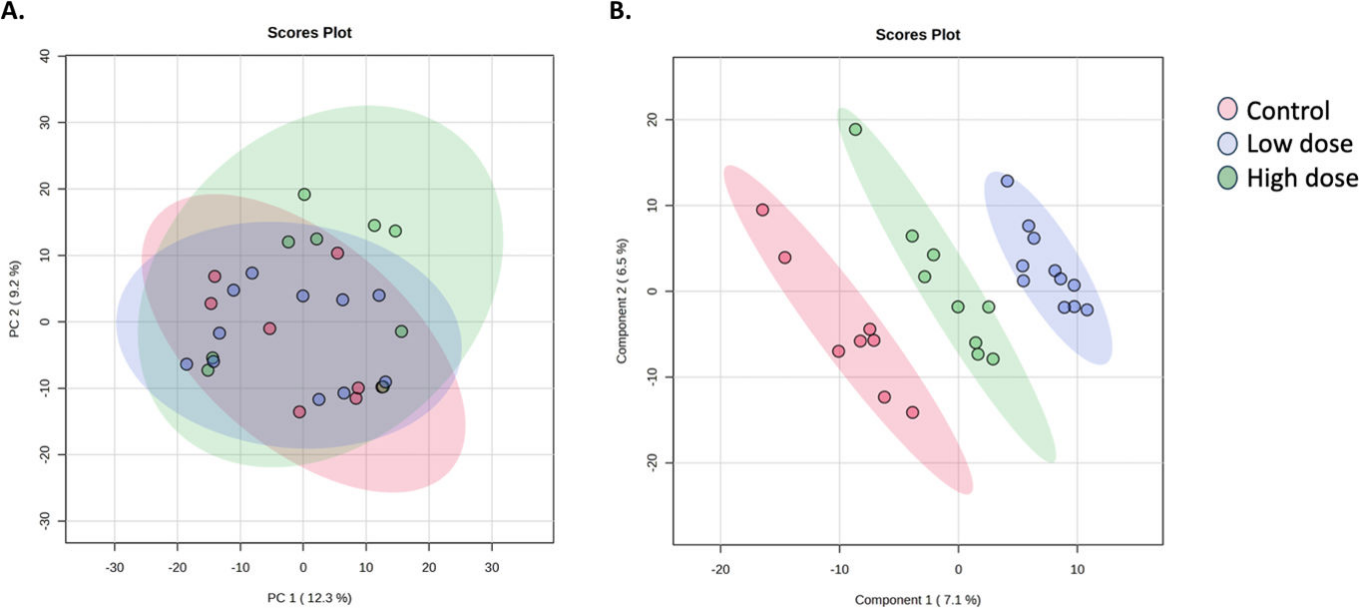
Analysis of the total cecal metabolomes of control, low-dose, and high-dose treatment groups with (**A**) an unsupervised principal component analysis model and (**B**) partial least squares discriminant analysis (PLS-DA).

To determine the metabolome similarities and dissimilarities between control and treated metabolomes (low-dose and high-dose ternary mixtures), a multi-group comparison of the total metabolomes was conducted. Multiple group analysis of the total metabolome as determined with principal component analysis highlighted distinct separation of the total metabolomes of the control group from the ternary mixtures (Fig. 3). Analysis of variance analysis (ANOVA) was subsequently conducted to investigate differences in metabolites among the treatment groups. ANOVA analysis detected 12 significant metabolites, of which Methylisopelletierine and Procarbazine were putatively identified (Table 4). Neither metabolite was detected in the annotated features list derived from Compound Discoverer (Table S8).

**TABLE 4:** Significant metabolites detected in ternary mixtures treatment groups as determined using ANOVA (Tukey’s post-hoc test; *P*-value ≤ 0.05)

*m/z_*RT(s)	Putative ID	F-value	*P*-value	−Log10(*P*)	FDR
175.1519__86	Not available	54.13	8.23e-10	9.08	1.64e-06
174.1486__103.39	Not available	52.67	1.08e-09	8.97	1.64e-06
192.1592__82.74	Not available	52.03	1.23e-09	8.91	1.64e-06
581.7156__145.79	Not available	51.61	1.33e-09	8.88	1.64e-06
174.1486__83.83	Not available	44.41	5.91e-09	8.23	5.83e-06
193.1625__82.74	Not available	43.15	7.82e-09	8.11	6.43e-06
156.1381__82.74	Methylisopelletierine	36.60	3.74e-08	7.43	2.63e-05
568.2721__125.15	Not available	28.86	3.19e-07	6.50	1.97e-04
244.1406__79.47	Procarbazine	25.55	9.05e-07	6.04	4.96e-04
555.2878__273.39	Not available	18.71	1.08e-05	4.97	5.31e-03
242.1464__611.08	Not available	15.65	3.92e-05	4.41	1.76e-02
366.9677__71.82	Not available	13.47	1.07e-04	3.97	4.39e-02

**Fig 4:**
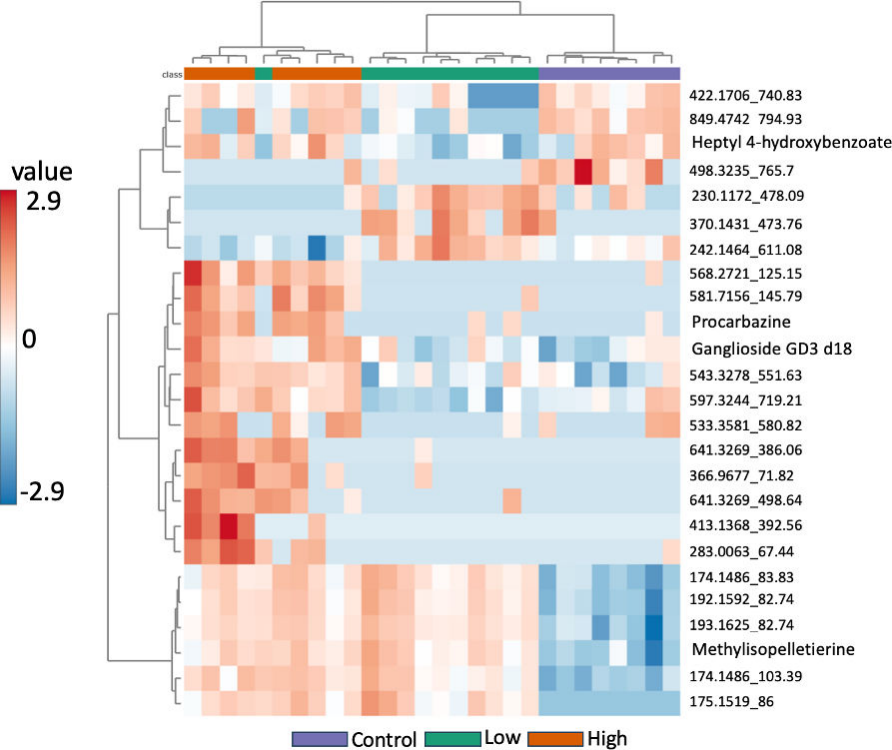
Hierarchical clustering analysis results of the top 25 metabolites detected in the control, low-dose, and high-dose groups. Colored boxes (red to blue) represent the normalized total ion intensity of metabolites within samples.

Hierarchical clustering was used to evaluate the cecal metabolomes for similarities and dissimilarities of detected dysregulated metabolites within each treatment group. We observed dose-response changes to the cecal metabolomes exposed to both ternary mixtures (Fig. 4). Putatively identified metabolites as determined by hierarchical clustering included Heptyl 4-hydroxybenzoate, Procarbazine, Ganglioside GD3 d18, and Methylisopelletierine (Fig. 4). Heptyl 4-hydroxybenzoate had a higher intensity in the control and high-dose mixture treatment groups (Fig. 4). Procarbazine and Ganglioside GD3 d18 both had a lower intensity in control and high-dose cecal metabolomes (Fig. 4). Both the low-dose and high-dose cecal metabolomes had a higher intensity of Methylisopelletierine (Fig. 4). A dose response was also detected for features, 230-1172_478.09, 370.1431_473.76, and 242.1464_478.611.08 (Fig. 4). Due to the mass spectrometry methods selected, nitrate could not be detected (Tables S8 and S9). However, atrazine, but not imidacloprid, was detected in the annotated peak list in Compound Discoverer, suggesting more rapid metabolism of atrazine (Table S8). In all, these results further highlight the significant difference in total ion intensity of individual metabolites between the treatment groups.

Functional analysis (*P*-value = 0.05, KEGG *Gallus gallus* library) was then conducted to assess pathway-level changes to the cecal metabolomes. Within MetaboAnalyst, significant hits refer to the number of empirical compounds in a data set detected in a specific metabolic pathway. The following metabolic pathways contained significant feature hits: metabolism of xenobiotics by cytochrome P450, amino sugar and nucleotide sugar metabolism, pyruvate metabolism, thiamine metabolism, and pantothenate and CoA biosynthesis (Table 5). To further understand the biochemical consequences of these shifts, pathway-level analyses were performed using both MetaCyc and BioCyc platforms to resolve functional changes in metabolite profiles and link them to taxonomically relevant genomes.

**TABLE 5:** Detected metabolic pathways derived from functional analysis comparing control, low-dose, and high-dose treatment groups (*P*-value = 0.05, KEGG *Gallus gallus* library)

Metabolic pathway	Hits (total)	Hits (significant)	AdjPFisher	AdjP EASE	AdjPGamma	Compound ID hits
Metabolism of xenobiotics by cytochrome P450	26	2	0.24	1	0.06	C14851; C14850; C14556; C14874; C14849
Amino sugar and nucleotide sugar metabolism	30	1	0.39	1	1	C03410
Pyruvate metabolism	4	1	0.24	1	1	C03451
Thiamine metabolism	1	1	0.08	1	1	C00378
Pantothenate and CoA biosynthesis	8	1	0.24	1	1	C00831

#### Agricultural groundwater contaminant mixture exposure redirects core cecal metabolic pathways toward xenobiotic processing and energy remodeling

**Fig 5:**
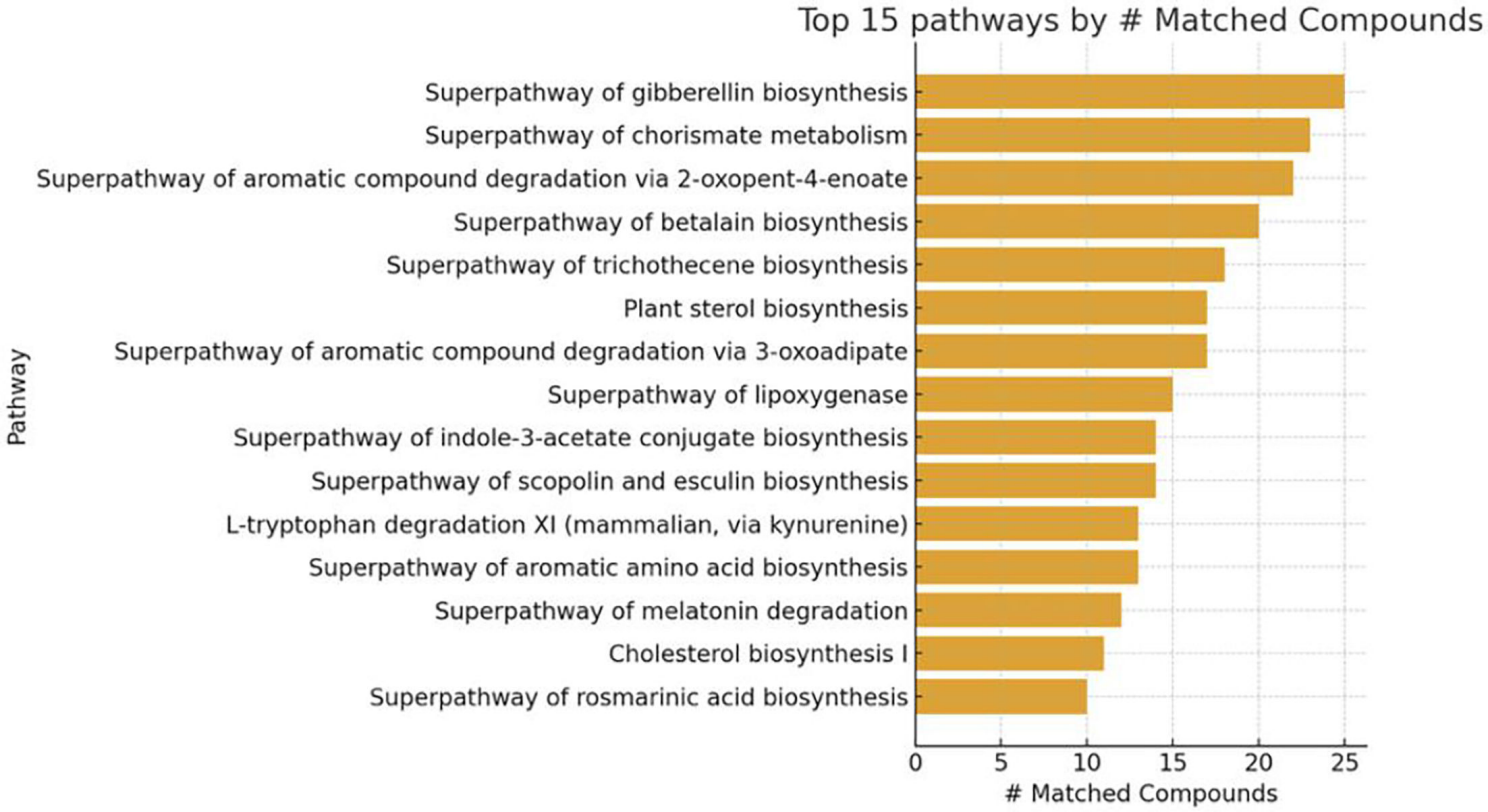
Top 15 metabolic pathways identified by the number of matched compounds using Metabolika analysis. Bar chart showing the top 15 enriched pathways ranked by the number of matched compounds across all analyzed samples. Each bar represents the total count of metabolites mapped to individual pathways. The most represented pathways include gibberellin biosynthesis, chorismate metabolism, and aromatic compound degradation via 2-oxopent-4-enoate, indicating strong activity in secondary metabolite and aromatic compound processing. These results highlight dominant biosynthetic and degradation processes contributing to overall metabolic shifts within the analyzed treatment groups.

Metabolika analysis of cecal metabolomes comparing control, low-dose, and high-dose treatment groups identified 15 significantly differentiated metabolic pathways (Fig. 5; Table S10). The number of matched compounds ranked these pathways. The superpathways of gibberellin biosynthesis and chorismate metabolism each contained the highest number of matched compounds (*n* = 23), followed by the superpathway of aromatic compound degradation via 2-oxopent-4-enoate (*n* = 22). Other prominent pathways included the superpathways of betalain biosynthesis (*n* = 20), trichothecene biosynthesis (*n* = 18), and aromatic compound degradation via 3-oxoadipate (*n* = 17). Additional plant-like and aromatic biosynthetic routes, such as plant sterol biosynthesis (*n* = 17), indole-3-acetate conjugate biosynthesis (*n* = 14), and jasmonoyl-amino acid conjugate biosynthesis (*n* = 9), were also represented. Lower-ranking pathways included melatonin degradation, cholesterol biosynthesis, and rosmarinic acid biosynthesis. Altogether, the top 15 enriched pathways were dominated by aromatic compound turnover, lipid oxidation, and secondary metabolite biosynthesis, indicating that cecal metabolomes were strongly characterized by oxidative and aromatic processing pathways typical of microbial and plant-derived metabolites. To complement these global pathway profiles, we next examined how specific taxa contributed to the observed metabolic remodeling by linking metabolite signatures to representative genomes using BioCyc pathway mapping.

#### Dose-dependent activation of biosynthetic, degradative, and energy pathways in the cecal metabolome

MetaCyc functional quantification revealed treatment-specific shifts in metabolomic activity (Tables S11 to 14). Overall, total pathway intensity increased progressively from control to high dose, indicating broad metabolic activation under chemical exposure. Within biosynthetic categories, fatty acid/lipid synthesis (FA/Lip Syn), secondary metabolite synthesis (Sec Metab Syn), and cofactor synthesis pathways were consistently more abundant in both low- and high-dose groups than in the control. The most significant differences were observed in amine and carbohydrate synthesis, aromatic compound synthesis, and metabolic regulation, all of which followed a clear dose-dependent pattern. In contrast, tetrapyrrole and cell-structure synthesis pathways peaked in the low-dose group, suggesting that structural and pigment biosynthesis were preferentially modulated at submaximal exposure levels.

Degradation pathways exhibited parallel dose-related patterns. Fatty acid/lipid degradation (FA/Lip Deg), amino acid degradation (AA Deg), carbohydrate degradation (Carbo Deg), and aromatic degradation (Aromatic Deg) were the most abundant categories across all treatments. Polymer degradation was highest in the low-dose group, while carbohydrate and chlorine degradation peaked under high-dose exposure. Energy-associated pathways showed pronounced treatment-linked variability: glycolysis, the pentose phosphate pathway (PPP), CO₂ fixation, and fermentation were all elevated in the high-dose group compared with the control, indicating heightened carbon flux and metabolic energy turnover. Conversely, tricarboxylic acid (TCA) cycle pathways were more active in the low-dose group, suggesting alternative energy flow routing at moderate exposure levels.

Aerobic and anaerobic respiration pathways also increased in both low- and high-dose groups, with hydrogen (H₂) production and photosynthesis-linked reactions detected exclusively in the exposed treatments and absent in the control group. This pattern suggests that microbial communities under chemical exposure developed metabolic traits that favor enhanced redox cycling and energy capture. Additionally, detoxification pathways, as well as macromolecular modification and acetylation/inactivation processes, were more active under high-dose conditions. Overall, metabolic activity followed the trend high > low > control, with lipid, aromatic, and cofactor metabolism contributing most prominently to the treatment-induced shifts in cecal metabolic potential.

#### Taxon-specific metabolic shift revealed differential susceptibility to chemical mixture exposure stress

To evaluate how metabolite production patterns corresponded with functionally relevant microbial taxa, annotated metabolites were mapped to representative genomes within the BioCyc database (Table S15). Differentially abundant taxa were first identified by 16S rRNA amplicon sequencing at the genus or family level, based on significant log-fold changes in relative abundance across treatments. For each of these taxa, a representative genome was subsequently selected in BioCyc that was closely related to poultry-associated isolates or known commensals of the avian ceca to ensure ecological relevance.

Among the identified groups, *Synergistes* and *Desulfovibrio* were most abundant in the control microbiome, with *Synergistes jonesii* 78-1 and *Desulfovibrio vulgaris* RCH1 chosen as the representative genomes due to their well-characterized anaerobic metabolism and prevalence in poultry intestinal environments. In contrast, members of the *Coriobacteriaceae* family were enriched by approximately 2 logs in both low- and high-dose treatments. They were represented by the *Coriobacteriaceae bacterium* 68-1-3, a genome isolated from intestinal environments with similar metabolic characteristics. These taxa were thus selected to represent control- and treatment-associated community profiles, respectively.

The absolute peak abundances of annotated compounds were compared between treatments and the control to assess metabolite-level shifts. Additionally, taxa enriched by more than 1 log in the exposed groups—Ruminococcus *torques* AM22-16 and *Akkermansia muciniphila* ATCC BAA-835—were included in the mapping to capture broader treatment-associated functional responses. The BioCyc framework integrated total metabolite peak intensities associated with each representative genome and summarized the top 25 metabolic pathways per treatment.

#### *Desulfovibrio* exhibited purine and cofactor remodeling after agricultural chemical mixture exposure

BioCyc pathway mapping of metabolites to the *D. vulgaris* representative genome revealed distinct metabolic activation patterns across treatments. Between the control and high-dose treatments, the most pronounced differences were observed in guanine and purine-associated pathways. The top five pathways exhibiting the highest differential pathway perturbation scores in the high-dose group (DPPS, ΔPPS) were guanosine nucleotide degradation III (ΔPPS = 32.8), purine nucleosides degradation I (ΔPPS = 25.6), and guanine and guanosine salvage I and II pathways (ΔPPS = 23.2, PPS = 23.2, respectively) (Table S15). While most pathways were enriched in the high-dose group, pathways associated with amino acid biosynthesis resulted in a small decrease under high exposure, including L-tryptophan, L-phenylalanine, and L-tyrosine biosynthesis (ΔPPS < 2) (Table S15). Comparative analysis between the control and low-dose groups revealed more modest, yet targeted, metabolic adjustments. The top pathways with the highest ΔPPS included hydroxymethylpyrimidine salvage (ΔPPS = 8.78), 4-amino-2-methyl-5-diphosphomethylpyrimidine biosynthesis I (ΔPPS = 8.78), purine ribonucleosides degradation (ΔPPS = 6.89), thiamine diphosphate biosynthesis I (ΔPPS = 5.55), and the superpathway of thiamine diphosphate biosynthesis (ΔPPS = 4.17) (Table S15). PPS values were higher in the control than in the low-dose group, suggesting mild suppression of nucleotide and cofactor biosynthesis at lower exposure levels. Contrary to pathways associated with selenate reduction, arsenate detoxification II (glutaredox), biotin-carboxyl carrier protein assembly, heme b biosynthesis III (from siroheme), reactive oxygen species degradation, 5-aminoimidazole ribonucleotide biosynthesis I, and ammonia assimilation cycle III were slightly higher in the low-dose group compared to the control (ΔPPS < 2). Collectively, these results indicate that *D. vulgaris* adapts to chemical exposure primarily through enhanced purine turnover and redox-associated metabolism, enabling energy maintenance and redox balance under stress.

#### *Ruminococcus* and *Akkermansia* increased glycolytic and redox activity after agricultural groundwater contaminant mixture exposure at the high dose

Both *Ruminococcus torques* AM22-16 and *Akkermansia muciniphila* ATCC BAA-835 exhibited dose-dependent activation of glycolytic and oxidative metabolic functions in the low- and high-dose treatment groups. In *R. torques*, the most strongly upregulated pathways included purine ribonucleoside degradation (ΔPPS = 25.9), guanine and guanosine salvage I and II (ΔPPS = 23.5), and trehalose degradation (low osmolarity) (ΔPPS = 13.1), indicating accelerated nucleotide turnover and enhanced redox cycling under high-dose exposure (Table S15). Complementary increases were observed in glycogen degradation II, glycolysis III (from glucose), homolactic fermentation, and UDP-N-acetyl-D-glucosamine biosynthesis I, where PPS values in the high-dose group nearly doubled relative to controls (Table S15). In contrast, the low-dose group showed no enrichment of these top pathways and exhibited slightly lower PPS values than the control, suggesting that mild exposure may suppress baseline energy and purine metabolism prior to high-dose induction.

In *A. muciniphila*, metabolic activation centered on carbohydrate and cofactor biosynthesis pathways. The highest pathway potential scores were associated with myo-inositol biosynthesis (ΔPPS = 13.1), GDP-mannose biosynthesis (ΔPPS = 7.2), glycolysis I (from glucose 6-phosphate) (ΔPPS = 5.7), and N-acetylglucosamine degradation I (ΔPPS = 4.7) (Table S15). The D-mannose degradation (ΔPPS = 5.2) was elevated in both exposure groups and absent in the control, while myo-inositol biosynthesis and glycolysis I (from glucose 6-phosphate) were notably higher in the high-dose group and reduced in the low-dose group compared to the control (Table S15). Smaller increases were also observed in amino acid and chorismate biosynthesis I pathways in the high-dose group. Collectively, these results demonstrate that *R. torques* and *A. muciniphila* respond to chemical stress through coordinated remodeling of carbohydrate, nucleotide, and cofactor metabolism, reflecting a dose-dependent shift toward enhanced energy generation and redox homeostasis under microplastic exposure.

#### *Coriobacteriaceae* and *Fournierella* prioritized nucleotide salvage and cell-envelope precursors upon agricultural groundwater contaminant mixture exposure

In *the Coriobacteriaceae bacterium* 68-1-3, BioCyc mapping revealed strong activation of nucleotide and cell-envelope biosynthetic pathways under high-dose conditions. The top responsive pathways included guanine and guanosine salvage pathways (ΔPPS = 23.32), while the rest of the pathways were less differentiated and included UDP-N-acetyl-D-glucosamine biosynthesis I, UDP-N-acetylmuramoyl-pentapeptide biosynthesis I (meso-diaminopimelate containing), and superpathway of N-acetylglucosamine, N-acetylmannosamine, and N-acetylneuraminate degradation (ΔPPS > 2) (Table S15). Additional pathways such as glycolysis (from glucose 6-phosphate), and PPP (non-oxidative branch) I, L-tryptophan biosynthesis, and 3-dehydroquinate biosynthesis I (ΔPPS > 1) were also moderately elevated, implying redirected carbon flux to support nucleotide and cell-surface precursor biosynthesis (Table S15).

Similarly, *Fournierella massiliensis* AM2 displayed strong activation of purine nucleoside degradation I (ΔPPS = 25.89), guanine and guanosine salvage I and II pathways (ΔPPS = 23.47), pseudouridine degradation (ΔPPS = 16.84), adenine and adenosine salvage III and adenosine nucleotides degradation II (ΔPPS > 13), trehalose degradation (low osmolarity) (ΔPPS = 13.09), glycogen degradation II (ΔPPS = 8.27), glycolysis III (from glucose) (ΔPPS = 7.18), homolactic fermentation, and UDP-N-acetyl-D-glucosamine biosynthesis I (ΔPPS > 6) (Table S15). Several other pathways were also perturbed (Table S15). While these pathways were enriched under the high-dose exposure, the same functions were reduced in the low-dose group relative to control, indicating a threshold-dependent metabolic response.

Collectively, both taxa exhibited a metabolic phenotype characterized by enhanced purine turnover, carbohydrate degradation, and envelope precursor biosynthesis, consistent with an adaptive reallocation of carbon and nitrogen toward energy and structural maintenance under chemical stress.

#### Integrated microbial community-level response to ternary chemical exposure

Exposure to agricultural chemicals induced coordinated metabolic reorganization across cecal microbial taxa, marked by enhanced energy production, redox cycling, and nucleotide turnover. Metabolika and BioCyc analyses showed dose-dependent activation of purine degradation, glycolysis, and cofactor pathways, reflecting metabolic flux redirected toward ATP generation and redox stabilization under chemical stress. *D. vulgaris* and *S. jonesii* primarily remodeled purine and pyrimidine metabolism, whereas *R. torques* and *A. muciniphila* activated glycolytic and carbohydrate-driven energy pathways. In contrast, *Coriobacteriaceae* and *Fournierella* emphasized nucleotide salvage and envelope-precursor biosynthesis, indicating structural adaptation and metabolic compensation. Together, these findings reveal a hierarchical, dose-dependent response in which low-level exposure transiently suppresses biosynthesis. At the same time, high-dose treatments trigger broad activation of catabolic, redox, and detoxification pathways, highlighting the community’s shift from growth-oriented metabolism toward energy conservation and chemical resilience. To determine whether similar or distinct functional patterns emerged under microplastic exposure, we next compared the control group to birds exposed to PE fiber MP.

### Effects of PE fiber microplastics on cecal microbiome and metabolome

#### Microplastics shift community composition without altering within-sample diversity

**Fig 6:**
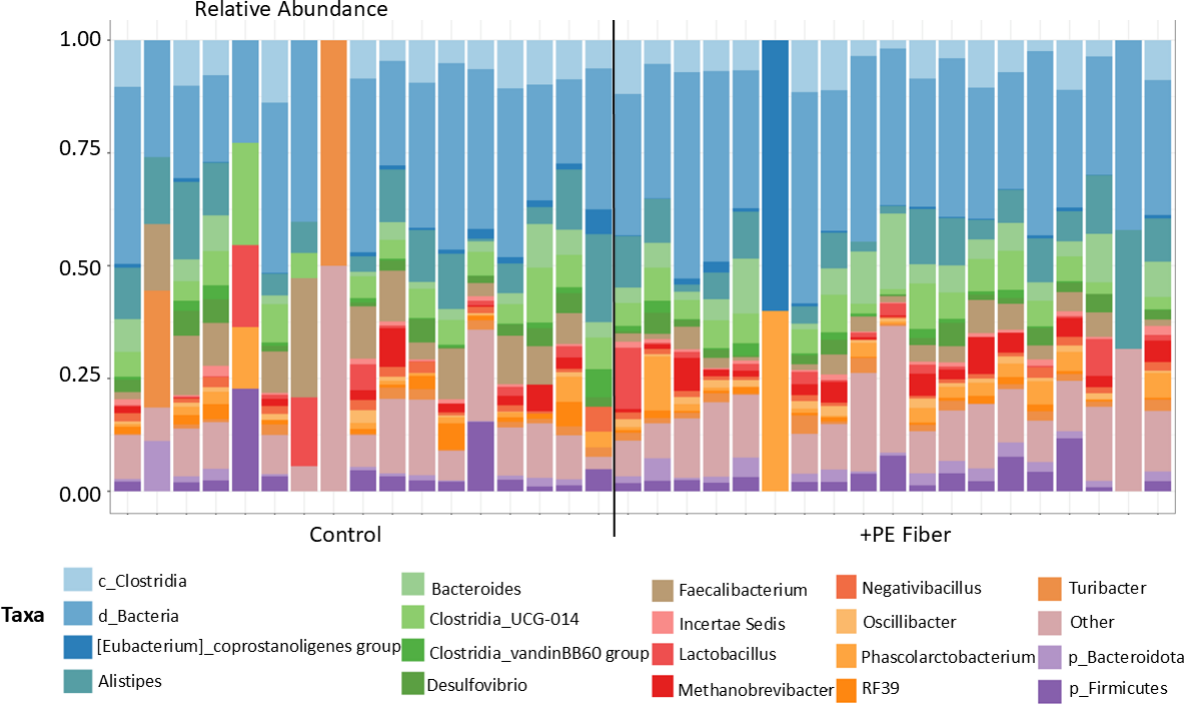
Relative abundance of the top 50 genera in each sample separated by treatment group (black line) for the +PE Fiber trial.

To assess the effect of PE fiber MPs on the cecal microbiome composition, the control (untreated) group was compared to the PE fiber MPs group. To assess this, 16S rRNA gene amplicon sequencing targeting the V4 region was conducted. The top 50 microbial taxa were plotted to the genus level (Fig. 6). Overall, there were significant differences between samples across the treatment groups (Fig. 6). The main effect “treatment” was not significant for α-diversity metrics, indicating that phylogenetic diversity within samples was not statistically different (Table S6) (Kruskal-Wallis pairwise, *P*-value ≥ 0.05, Q-value ≥ 0.05). However, overall β-diversity metrics comparing control to +PE Fiber were statistically significant as determined by PERMANOVA (Table S7) (*P*-value ≤ 0.05; Q-value ≤ 0.05). Thus, confirming phylogenetic differences between the control and +PE Fiber treatment groups. Overall assessment of dissimilarities among the treatment groups as determined by Bray-Curtis and Weighted UniFrac PCoA plots also demonstrated distinct clustering (Fig. S6).

**Fig 7:**
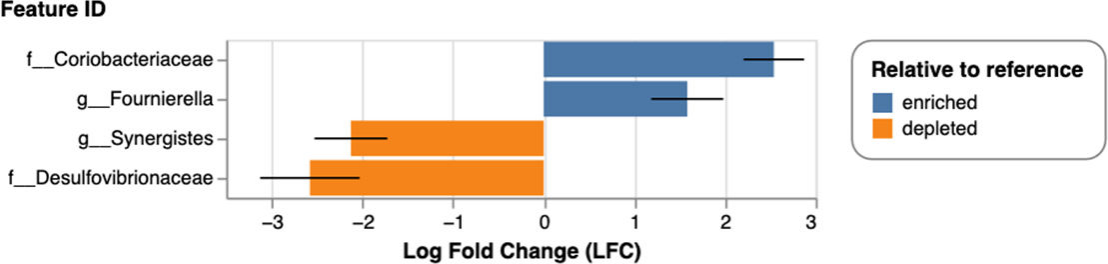
Differential abundance of genera in the +PE fiber group relative to the control group as determined by ANCOM-BC. Unclassified genera were annotated to the family level.

**Fig 8:**
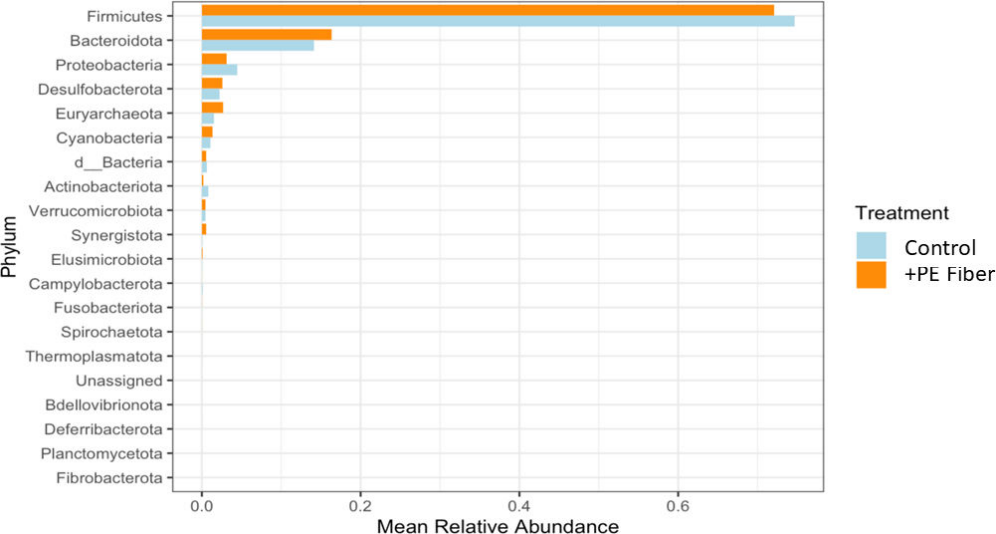
Comparison of mean relative abundance of phylum by treatment group for +PE fiber trial.

Differential abundance testing using ANCOM-BC was subsequently conducted to quantify changes in the relative abundance of microbial taxa as related to the control group. It was determined that the genus *Fournierella* and an unclassified genus in the family *Coriobacteriaceae* were enriched in the +PE Fiber treatment group (Fig. 7), while the genus *Synergistes* and one unclassified genus in the *Desulfovibrionaceae* family were depleted in +PE Fiber cecal microbiomes. We subsequently assessed microbial composition variability among control and +PE fiber treatment groups at the phylum level. Analysis of the mean relative abundance of phylum was used to evaluate potential dysbiosis in the cecal microbiomes. The mean relative abundance of *Firmicutes* was higher in the control group, while *Bacteroidota* was higher in the +PE fiber treatment group, indicating the +PE fiber treatment group had a lower *Firmicutes/Bacteroidota* ratio compared to the control treatment group (Fig. 8). These results also highlighted that the +PE fiber treatment group had a higher mean relative abundance of *Bacteroidota* and *Desulfobacterota,* while the control group exhibited a higher mean relative abundance of *Firmicutes* and *Proteobacteria* (Fig. 8). Given these microbial compositional changes, we next examined whether PE fiber exposure induced shifts in the cecal metabolome reflective of altered microbial or host metabolic processes.

#### PE fiber exposure produced broad dysregulation within the cecal metabolome.

**Fig 9:**
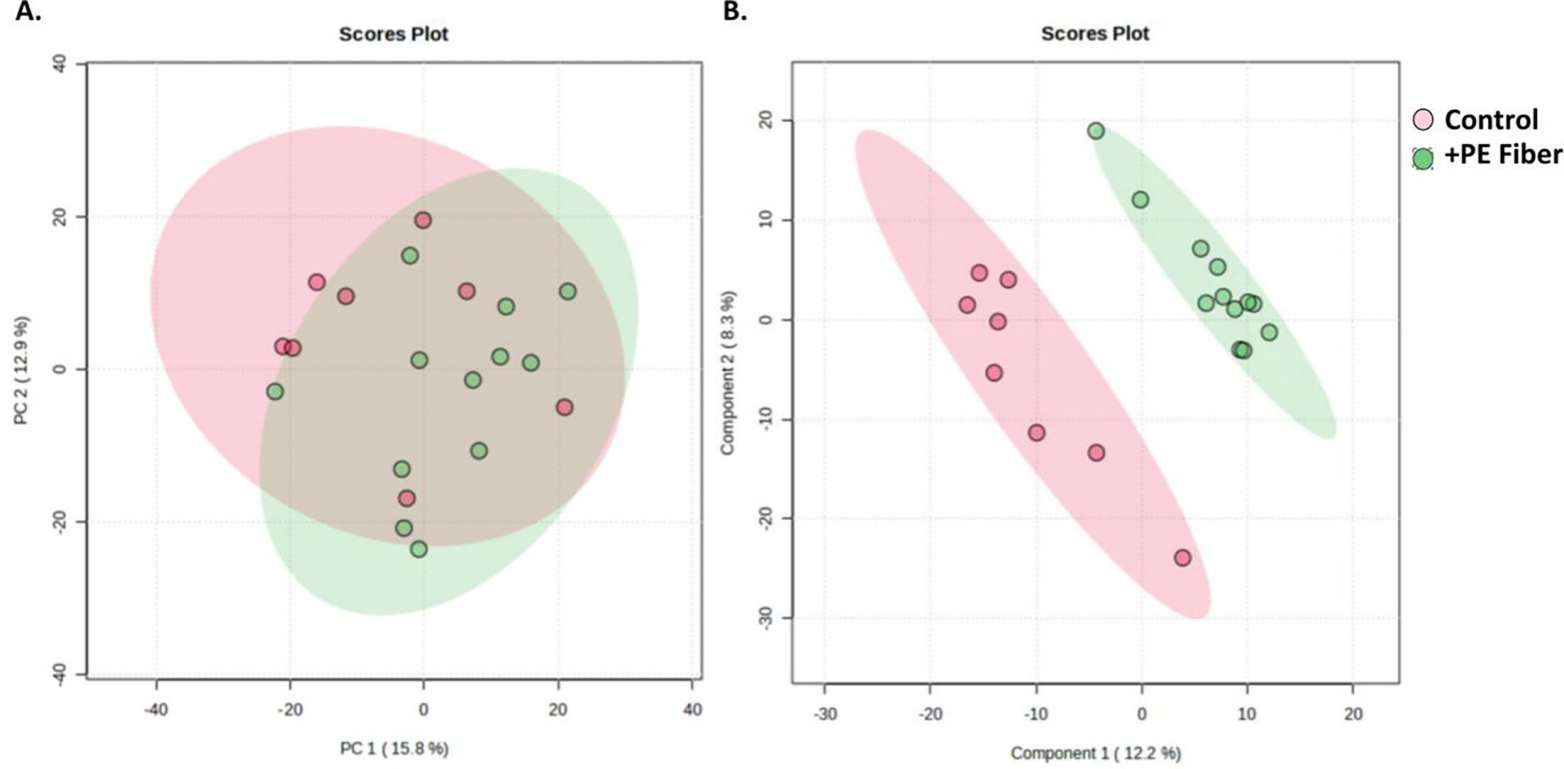
Analysis of the total cecal metabolomes of control and +PE fiber treatment groups with (**A**) an unsupervised principal component analysis model and (**B**) partial least squares discriminant analysis (PLS-DA).

To assess the impact PE fiber MPs have on the cecal metabolome, a pairwise comparison was conducted on the total metabolome of each group (i.e., control cecal metabolome vs +PE fiber cecal metabolome). Analysis of the total metabolome as determined with an unsupervised model, principal component analysis, and a supervised model, partial least squares discriminant analysis (PLS-DA), highlighted distinct clustering of the total metabolome of +PE fiber (Fig. 9). A summary of all detected metabolites is provided in Table S16. The results of pairwise comparisons indicated there were 113 metabolites that were significantly down-dysregulated, 73 significantly up-dysregulated, and 4,742 insignificant metabolites (Table S17). Volcano plot analysis was then conducted in Compound Discoverer, which highlighted that Vitamin C, Glucuronolactone, 2-arachidonoylglycerol, and dihydroxyphenylalanine were among the metabolites significantly down-dysregulated in the +PE fiber treatment group (Table S18) (Log2 fold change ≤ −2; *P*-value ≤ 0.05). However, associated metabolites for the control treatment group included uplandicine, ethyl docosahexaenoate, and methyl isoquinoline-3-carboxylate as annotated by Compound Discoverer (Table S18) (Log2 fold change ≥ 2; *P*-value ≤ 0.05). Overall, there were a greater number of metabolites dysregulated due to the presence of +PE fiber compared to the control. These results indicate that the presence of PE fiber significantly impacts cecal microbial activity.

**Fig 10:**
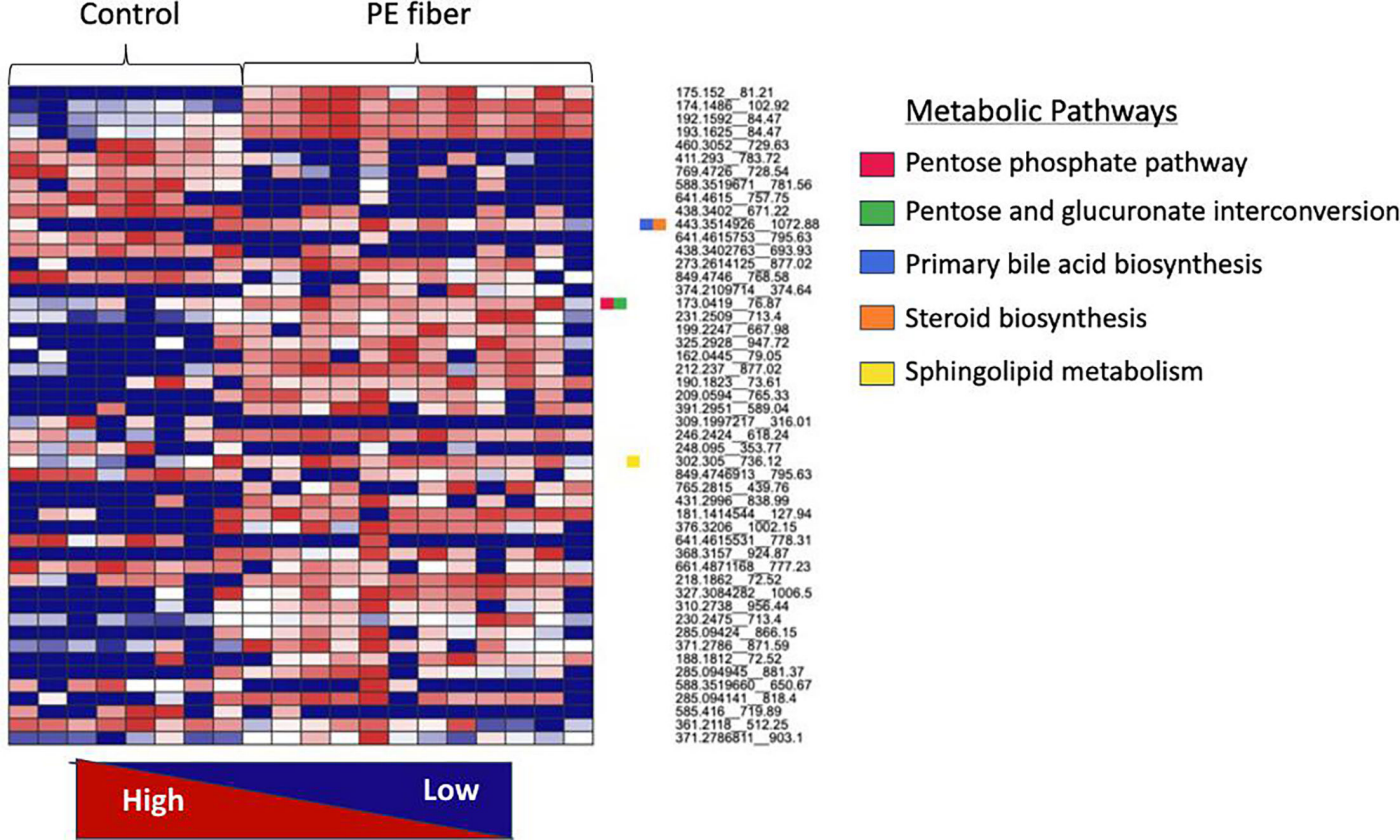
Pathway-level analysis performed in MetaboAnalyst 6.0 using the Function analysis module. Functional analysis incorporates a gene set enrichment and mummichog algorithm (*P*-value = 0.05, KEGG library *Gallus gallus* Library) for accurate detection of changes in total ion intensity of features.

Next, pathway-level changes in the +PE fiber treatment group were assessed using the functional analysis module in MetaboAnalyst. It was determined that there was significant modulation in metabolic pathways associated with the +PE fiber treatment group. Specifically, there was a higher total ion intensity of metabolites linked to the pentose phosphate pathway, pentose and glucuronate interconversion, primary bile acid biosynthesis, steroid biosynthesis, and sphingolipid metabolism (Fig. 10). In the +PE fiber metabolome, there was also a noticeably higher intensity of features 175.152_81.21 *m/z*_RT, 174.1486__102.92 *m/z*_RT, 192.1592_84.47 *m/z*_RT, and 193.1625_84.47 *m/z*_RT (Fig. 7). For each feature, the *m/z* represents mass to charge ratio, and RT is retention time in seconds for that metabolite feature. These metabolites could not be mapped to metabolic pathways. Despite this, the dysregulation of these metabolites further highlights that PE fiber MPs significantly impacted microbial activity. To interpret these metabolic perturbations in a functional context, we next examined global pathway enrichment and taxon-specific metabolic associations using MetaCyc and BioCyc databases.

#### Microplastics suppress oxidative metabolism and promote glycolytic compensation

MetaCyc pathway quantification of the +PE fiber and control metabolomes revealed distinct clustering of biosynthetic, degradative, and energy-linked functions (Tables S19 to 22). Overall, total pathway intensity was slightly higher in the PE fiber group, indicating that exposure to polyethylene microplastics restructured cecal microbial metabolism by selectively activating biosynthetic and cofactor-dependent processes. Within biosynthetic pathways, PE fiber microbiomes exhibited elevated activity in aromatic compound synthesis, cell-structure biosynthesis, metabolic regulation synthesis (MetabReg Syn), carbohydrate synthesis, amine synthesis, and amino acid synthesis. In contrast, nucleotide synthesis, tetrapyrrole synthesis, and cofactor/vitamin biosynthesis were reduced in the PE fiber group compared to controls (Table S19). This suggests that microplastic exposure redirected biosynthetic energy from nucleic acid and cofactor production toward structural, regulatory, and aromatic compound formation.

Across degradative categories, PE fiber metabolomes displayed moderate enrichment in amino acid degradation but lower intensities for all other major pathways, including carbohydrate, nucleotide, secondary metabolite, hormone/signal, aromatic compound, and chlorinated compound degradation (Table S19). These reductions imply that PE fiber exposure suppressed global catabolic turnover and reduced the community’s ability to degrade diverse organic substrates.

Energy metabolism further distinguished the two groups. Glycolysis was markedly elevated in the PE fiber microbiomes, indicating a compensatory increase in substrate-level phosphorylation and redox balancing under microplastic-induced stress (Table S21). Conversely, aerobic and anaerobic respiration, the PPP, the TCA cycle, CO₂ fixation, and chemoautotrophy were all higher in control samples. Together, these trends reveal a shift from efficient oxidative and fermentative energy metabolism toward simplified glycolytic dependence under PE exposure. Within the “Other pathways” category, the PE fiber microbiomes showed enrichment in acetylation/inactivation and macromolecule modification pathways, consistent with adaptation to and detoxification of xenobiotic stress (Table S22). In contrast, control samples displayed higher activity in inorganic nutrient utilization and C1 metabolism, indicating a more balanced nutrient cycling capacity under baseline conditions. Complementary metabolite analysis identified chorismate metabolism as one of the most strongly differentiated pathways between treatments (Fig. 5). Chorismate serves as a precursor for aromatic amino acids and secondary metabolites, and its upregulation in the PE fiber group supports the notion that microplastic exposure stimulates aromatic compound biosynthesis as a metabolic coping mechanism.

#### Taxa enriched in the control condition lose purine, sulfur, and cofactor pathways under PE fiber microplastics exposure

To assess how microplastic exposure altered the metabolic potential of taxa predominant in control microbiomes, metabolites were mapped to the *Synergistes jonesii* 78-1 representative genome within the BioCyc database. *Synergistes* were among the most abundant genera in the control group but were markedly depleted in the +PE fiber treatment. Pathway mapping showed that *S. jonesii* metabolism was primarily driven by nucleotide turnover, amino acid synthesis, and cofactor biosynthesis pathways (Table S23). Comparative quantification revealed that nearly all top-ranked pathways exhibited higher pathway potential scores (PPS) in the control microbiome. The most differentially reduced pathways under +PE Fiber exposure included L-alanine biosynthesis III (ΔPPS = 8.97), xanthine and xanthosine salvage (ΔPPS = 6.75), purine nucleoside degradation II (aerobic) (ΔPPS = 6.61), L-cysteine biosynthesis I (ΔPPS = 6.31), guanosine nucleotide degradation III (ΔPPS = 6.18), and iron–sulfur cluster biosynthesis (ΔPPS = 5.64) (Table S23). Additional pathways strongly suppressed under PE exposure involved purine-associated degradation, CoA biosynthesis (prokaryotic), and guanine/guanosine salvage, whereas others, such as adenosine nucleotide degradation, were less affected (ΔPPS < 3). In contrast, tRNA charging (ΔPPS = 3.78) and biotin–carboxyl carrier protein assembly (ΔPPS = 0.42) exhibited modest enrichment in the +PE fiber group.

Metabolite mapping to the *D. vulgaris* RCH1 representative genome revealed treatment-linked shifts consistent with the reduced abundance of this genus under PE fiber exposure (Table S23). Overall, PPS were consistently higher in the control group. The top pathways with the greatest differential pathway perturbation scores (ΔPPS) were L-alanine biosynthesis III (ΔPPS = 8.91), pyridoxal 5’-phosphate salvage I (ΔPPS = 8.81), biotin biosynthesis from 8-amino 7-oxononanoate I (ΔPPS = 7.26), xanthine and xanthosine salvage, L-cysteine biosynthesis I, and cytidylyl molybdenum cofactor sulfurylation (ΔPPS > 6). Other perturbed pathways included superpathway of pyridoxal 5’-phosphate biosynthesis and salvage, iron-sulfur cluster biosynthesis (ΔPPS > 5), with minor differences (ΔPPS < 4) observed for additional cofactor and nucleotide-related pathways (Table S23). Both *Synergistes* and *Desulfovibrio* showed the highest pathway intensities in the control group, with consistent depletion across amino acid, purine, cofactor, and L-cysteine and iron-sulfur metabolism under PE fiber exposure.

#### Microplastics-enriched taxa exhibit attenuated biosynthetic and energy pathways

Mapping of annotated metabolites to the *Coriobacteriaceae bacterium* 68-1-3 and *F. massiliensis* AM2 representative genomes revealed parallel patterns of metabolic adaptation to PE fiber exposure. Both taxa were enriched in +PE fiber group compared to control, indicating microfiber-induced shifts in metabolic potential. For *C. bacterium* 68-1-3, the highest differential pathway perturbation scores were observed in L-alanine biosynthesis III (ΔPPS = 8.92), pyridoxal 5′-phosphate salvage I (ΔPPS = 8.82), xanthine and xanthosine salvage (ΔPPS = 6.76), and L-cysteine biosynthesis I (ΔPPS = 6.31), with smaller differences (ΔPPS = 4–5) in iron-sulfur cluster biosynthesis, PPP (non-oxidative branch) I, and coenzyme A biosynthesis I (prokaryotic) (Table S23). Additionally, guanine and guanosine salvage I and II, tRNA charging, and molybdopterin biosynthesis (ΔPPS > 3) were depleted in +PE fiber compared to the control. Only a few routes, including pyridoxal 5′-phosphate salvage I and tRNA charging (ΔPPS = 3.78), were selectively enriched in +PE fiber.

Similarly, *F. massiliensis* AM2 exhibited depletion of most biosynthetic and degradation pathways under PE fiber treatment. The top differentiated pathways included L-alanine biosynthesis III (ΔPPS = 8.92), xanthine and xanthosine salvage, cytidylyl molybdenum cofactor sulfurylation, and L-cysteine biosynthesis I (ΔPPS > 6), along with minor differences in inosine 5′-phosphate degradation, bis(guanylyl molybdopterin) cofactor sulfurylation, coenzyme A biosynthesis (prokaryotic), and adenosine nucleotides degradation II (ΔPPS > 4) (Table S23). As in *Coriobacteriaceae*, these pathways were depleted in +PE fiber group, with only tRNA charging (ΔPPS = 3.78) and biotin-carboxyl carrier protein assembly (ΔPPS = 0.42) remaining upregulated in +PE fiber. These results indicate that while *Coriobacteriaceae* and *F. massiliensis* increased in abundance under PE fiber exposure, their intracellular metabolism exhibited broad attenuation of biosynthetic and energy-related functions.

### Microplastics-induced metabolic reprogramming of microbial community

Integrated MetaCyc and genome-resolved pathway analyses revealed clear, treatment-dependent restructuring of cecal microbial metabolism in response to PE fiber exposure. At the community level, PE fiber metabolomes displayed increased overall pathway intensity, reflecting selective activation of biosynthetic and glycolytic functions alongside diminished oxidative and degradative capacity. Pathways supporting aromatic compound synthesis, structural biomolecule formation, and amino acid biosynthesis were most elevated, whereas nucleotide, tetrapyrrole, and cofactor/vitamin synthesis pathways were consistently reduced.

Taxon-specific mapping further resolved opposing metabolic trends between control- and PE-associated populations. Control-enriched *Synergistes* and *Desulfovibrio* exhibited the highest PPS for purine, sulfur, and cofactor metabolism, with substantial depletion of these functions under PE fiber exposure. In contrast, *Coriobacteriaceae* and *Fournierella* were abundant in the +PE fiber group and exhibited an overall attenuation of biosynthetic and energy-linked pathways. Both taxa retained selective upregulation of tRNA charging and pyridoxal 5′-phosphate salvage, indicating limited preservation of translational and cofactor-recycling capacity. These results describe a functional transition from diverse oxidative and anabolic metabolism in control microbiomes to simplified, fiber-adapted metabolic states dominated by glycolytic and structural biosynthetic activity under PE fiber exposure.

## DISCUSSION

### Environmentally relevant contaminant exposures remodel gut microbial community and metabolism, but do not cause detectable tissue damage

In the current study, we aimed to characterize how a 42-day exposure to a ternary mixture of nitrate, atrazine, and imidacloprid or PE fiber microplastics affects broiler performance, epithelial integrity, and cecal microbiome and metabolome profiles. It has been well established that microplastics cause adverse health effects across various species (54–57). However, their impact on terrestrial food-producing animals, particularly poultry, remains poorly characterized despite increasing environmental detection. Poultry represents an essential species for assessing exposure, given their close interactions with feed, litter, and groundwater sources that agricultural and emerging pollutants may contaminate. Because these compounds may persist and bioaccumulate within avian tissues and their uptake during production also poses potential implications for human exposure through the poultry food chain. Agricultural chemicals are continually introduced into waterways through atmospheric deposition, runoff, and erosion (58, 59). Their persistence in groundwater has raised concerns, as concentrations exceeding regulatory standards are increasingly reported (24, 60). To address these environmentally relevant risks, we designed exposures to reflect realistic field concentrations and potential high-end scenarios. The low-dose mixture (35,000 ppb nitrate + 1.7 ppb atrazine + 0.58 ppb imidacloprid) paralleled concentrations measured in the 2021 Wisconsin DATCP Targeted Sampling Report (24), whereas the high-dose mixture (100,000 ppb nitrate + 3,000 ppb atrazine + 3,000 ppb imidacloprid) was selected to evaluate potential effects at concentrations observed to impact microbial growth *in vitro*. Ultimately, these compounds were chosen based on their frequent co-detection in agricultural groundwater and complementary toxicological profiles, which together represent a realistic environmental exposure scenario.

Overall, there were no overt signs of toxicity or treatment-related pathology among broilers, although significant differences in BWG and FI were observed across both contaminant types (Tables 2 and 3). This pattern aligns with previous findings that agricultural contaminants may not immediately induce clinical toxicity but can still interact with host physiology. Foster & Khan (61) reported that laying hens exposed to 100 ppm atrazine showed no visible physiological damage. However, atrazine residues persisted in feces for up to four days after exposure, supporting the potential for xenobiotic persistence and bioaccumulation in gastrointestinal tissues. Our previous work demonstrated that exposure to the same low-dose mixture, as well as related ternary combinations, significantly reduced growth kinetics of poultry cecal microcosms and epithelial cell viability in Caco-2 models (30), suggesting that synergistic chemical-biological interactions may occur even below acute toxicity thresholds. Consistent with these findings, the present *in vivo* study revealed that exposure to both low- and high-dose ternary mixtures resulted in notable shifts in cecal microbial community composition, including enrichment of five genera not detected in control birds (Fig. 2). Metabolomic profiles further confirmed that these chemical mixtures restructured microbial activity and metabolic network organization. Such congruence between taxonomic and metabolic outcomes reinforces that chronic exposure to realistic contaminant mixtures can alter intestinal functional capacity without inducing overt histopathological changes. It is important to acknowledge that heat stress and wing-band injuries, which occurred in both control and treated groups before and during the trials, could potentially influence microbial and metabolomic profiles. However, given the treatment-specific shifts observed in the cecal microbiome and metabolome of the +PE fiber, low-dose, and high-dose groups, the impact of these background variables is likely minimal.

These results highlight a central concept emerging from this study, functional remodeling rather than structural damage, as the unifying signature of contamination. These findings underscore the importance of microbiome and metabolome assessments as sensitive indicators of subclinical environmental impacts, capable of revealing early adaptive or compensatory shifts before pathology becomes detectable. The following discussion examines these microbial compositional responses in greater detail to clarify how community-level and taxon-specific dynamics emphasize these broader functional transitions.

### Contaminant exposure reshapes cecal microbial community structure without diversity loss, triggering metabolic functional capability instability

Cecal microbial diversity responded in a dose-dependent manner to contaminant classes, highlighting distinct modes of ecological disruption. For birds exposed to a low-dose ternary chemical mixture and the PE fiber group, α-diversity metrics did not show significant differences from the control (Fig. S6). On the contrary, a high-dose group resulted in significantly different diversity compared to control, indicating a dose-dependent separation among treatment groups (Fig. S6). Beta diversity analyses revealed significant differences in community structure between ternary mixtures and PE fiber groups relative to controls, supporting compositional restructuring even at the environmentally relevant low-dose mixture (Fig. S6). Together, these trends suggest that while under low doses and PE fiber, microbial diversity remains unchanged, communal changes are evident in structural differences that become pronounced at higher doses.

Microplastics are increasingly recognized as drivers of gut dysbiosis, including the loss of beneficial commensal bacteria (62). In our prior *in vitro* cecal mesocosm experiment, PE fiber exposure similarly shifted community structure, reducing *Firmicutes* relative to *Bacteroidota* (49). In the current study, differential abundance analysis revealed enrichment of *Fournierella* and depletion of *Synergistes* in the +PE fiber group. These taxa are also responsive to prebiotic carbohydrate inputs, providing a useful comparison with positive-control systems such as fructooligosaccharides (FOS). Asare et al. (22) reported that PolyFermS cultures supplemented with FOS increased the abundance of *Fournierella*, while Jin et al. (63) associated elevated *Fournierella* with altered calcium availability. In this context, FOS restructures the microbiota to enhance fermentation and nutrient utilization. In contrast, birds exposed to PE fibers exhibited lower BWG and FI at D28 than controls (Table 2), suggesting potential nutrient malabsorption or altered appetite regulation. Given FOS-driven microbial changes typically support coordinated cross-feeding and improved energy harvest (56, 57), the similarity between PE fiber- and FOS-associated taxa suggests that microplastics may dysregulate the same metabolic pathways prebiotics aim to optimize. Rather than promoting efficient fermentation, PE fiber exposure may destabilize microbial energy networks, ultimately reducing growth efficiency.

Functional evidence of this restructuring was reflected in perturbations of metabolic pathways based on *Gallus gallus* model. For both contaminant types, a significant modulation of pantothenate and CoA biosynthesis, pyruvate metabolism, and thiamine metabolism was observed (Table 5). These patterns are also reported in amphibian gut microbiomes following atrazine exposure (64). In this study, pantothenate and thiamine pathways exhibited significant feature hits, indicating active disruption of B-vitamin-linked metabolic networks. Pantothenate (vitamin B5) and thiamine (vitamin B1) are critical cofactors for microbial and host oxidative metabolism (65), immune function, and cellular energy regulation (66). Poultry cannot synthesize thiamine and depend entirely on dietary sources (67), making its depletion within metabolomic profiles noteworthy. Reduced availability or increased microbial utilization of these cofactors could impair carbohydrate, protein, and lipid turnover, contributing to the reduced feed efficiency observed in exposed birds on some days. Thus, chronic low- or high-dose exposure to nitrate--atrazine-imidacloprid mixtures can disrupt foundational metabolic pathways essential for host energy balance. Additionally, these findings support the idea that significant functional disruption can occur without epithelial damage (68). These taxonomic and metabolic patterns also support the silent dysbiosis idea (69) under PE fiber and low dose conditions. While the taxa and metabolic pathways adapt to impair nutrient turnover and redox homeostasis, the community membership remains relatively stable. Therefore, to determine whether these shifts translated into altered metabolic capacity, we next examined pathway-level changes, focusing on global changes (Metabolomika and MetaCyc) and taxon-linked BioCyc functional profiling.

### Chemical mixtures activate a detoxification-oriented, energy-conserving metabolic state

Across both contaminant doses, chemical exposure induced clear metabolic shifts of the cecal ecosystem. Taxa associated with aromatic compound turnover and carbohydrate fermentation, *Fournierella*, *Coriobacteriaceae*, and *Ruminococcus,* were enriched in all exposed groups, indicating enhanced catabolic activity under xenobiotic stress. In contrast, *Desulfovibrio,* which contributed to short-chain fatty acid (SCFA) production, sulfur metabolism, and redox regulation, was depleted. Global metabolomic profiling revealed broad pathway enrichment across both ternary mixture groups (Table S10). Although these profiles reflect a mixture of host-derived and microbially derived metabolites, increased activity in chorismate metabolism, aromatic compound degradation, and nucleotide salvage pathways indicates elevated metabolic flux through the shikimate branch, a hallmark of redox compensation during xenobiotic stress (70). Enrichment in L-tryptophan degradation XI (via kynurenine) suggests host involvement, consistent with early oxidative stress and immune signaling (71). KEGG *Gallus gallus* analysis supported this interpretation, identifying enrichment of xenobiotic metabolism pathways (Table 5), central to hepatic and intestinal detoxification and energy balance (72, 73). Together, these findings reflect a coordinated microbial-host response involving oxidative detoxification, cofactor regeneration, and alternative electron-flow routing. Such responses have been described as central features of microbial resilience, in which communities increase cofactor turnover, redistribute electron flow, and activate alternative detoxification enzymes to maintain metabolic stability (74).

At low-dose exposure, reduced activity of nucleotide and cofactor-linked pathways (including tetrapyrrole biosynthesis) and mild suppression of aerobic respiration suggest a resource-conservation strategy that maintains oxidative metabolism and community structure. This pattern parallels reports that atrazine suppresses cofactor-dependent metabolism and alters redox balance without overt structural disruption (75, 76). Similar functional remodeling has also been observed in soil microbial communities exposed to atrazine. Fernandes et al. (64) observed an increase in degradation gene markers without major shifts in overall community structure. Similarly, in the current study, no significant differences were observed in microbial diversity and community structure at low-exposure conditions compared to control (Table S4). Increased activity in TCA cycle intermediates, hydrogen production, and cell-structure biosynthesis at low dose (Tables S11 and S13) indicates selective modulation rather than broad functional collapse (65) and suggests that oxidative metabolism can remain dominant until the chemical threshold is reached. In contrast, the high-dose ternary mixture resulted in broad enrichment of glycolysis, fermentation, CO₂ fixation, PPP, carbohydrate, and aromatic compound synthesis (Tables S11 and S13). Activation of these pathways may indicate enhanced carbon flux, alternative energy routing, redox modulation, and detoxification under more severe chemical pressure, aligning with metabolic reprogramming documented in pesticide-exposure gut ecosystems (66). Increased acetylation and inactivation pathways further indicate enhanced xenobiotic biotransformation (Table S14), consistent with atrazine-responsive nitrogen and energy metabolism in soil microbiomes. Atrazine clearance was associated with microbial functions associated with nitrogen metabolism and energy generation in soil microbiome (67).

The dose-dependent differences show that low-dose exposure downregulated energetically costly biosynthetic pathways while maintaining oxidative metabolism. In contrast, high-dose exposure triggered a shift toward glycolytic flux, fermentation, and detoxification. Global metabolic activity followed the trend high > low > control, driven primarily by lipid, aromatic, and cofactor metabolism. Both doses induced an energy-redox compensation phenotype in which microorganisms redirected carbon and electron flow to sustain growth under xenobiotic stress. Increased aerobic and anaerobic respiration pathways, along with dose-specific hydrogen production and photosynthesis-linked reactions (Table S13), further reflected enhanced redox cycling and energy mechanisms. This pattern aligns with observations that pesticide-stressed microorganisms can transition to anaerobic respiration and alternative electron sinks to maintain redox balance when oxidative metabolism is limited (66, 72). This compensatory metabolism may explain how low-dose exposure produced significant functional restructuring without detectable changes in overall taxonomic diversity.

The taxonomic shift followed a similar trend. *Desulfovibrio* was depleted in the chemical-mixture groups (Fig. 2). In contrast, *Ruminococcus* and *Akkermansia* were enriched, alongside activation of glycolysis, carbohydrate degradation, and cofactor biosynthesis pathways, suggesting increased carbohydrate-driven energy production and cofactor regeneration to balance detoxification stress. *Coriobacteriaceae* and *Fournierella* also increased with enriched pathways for nucleotide salvage and envelope-precursor biosynthesis under high-dose exposure. Together, these shifts indicate that chemical exposure may selectively disfavor sulfate-reducing taxa while favoring microorganisms capable of redirecting carbon and electron flow toward energy generation and repair. To determine if a similar compensatory strategy occurred under microplastic exposure, we next examined taxonomic and functional responses in tceca of birds treated with PE fiber MPs.

### Microplastics induce a minimal-energy, glycolysis-driven survival mode

Global metabolomic analysis revealed that PE fiber exposure induced a metabolic phenotype more similar to the low-dose chemical mixture than the high-dose response. Chorismate metabolism was the only significantly differentiated global pathway, indicating that microplastic exposure elicits selective, rather than broad remodeling (Fig. 5). Similarly to the response of agricultural contaminants, its enrichment suggests a metabolic shift toward aromatic compound biosynthesis as a coping mechanism under polymer exposure (77). Functional analyses indicated pronounced shifts in energy- and lipid-associated pathways. KEGG *Gallus gallus* analysis indicated modulation of the PPP, pentose and glucuronate interconversion, bile acid biosynthesis, steroid biosynthesis, and sphingolipid metabolism. Similar effects have been observed in oysters exposed to PE (78) and in mice exposed to polystyrene MPs, while bile acid disruption and intestinal damage were reported (79). Because sphingolipids regulate membrane integrity and ceramide accumulation, which has been linked to metabolic disease (62, 63, 68), these findings suggest that PE fibers may alter hepatic signaling and nutrient absorption over prolonged exposure (70–73). Although the overall global metabolic intensity changed only modestly, the distribution of pathway types demonstrated selective activation of biosynthesis (aromatic compound, structural, carbohydrate, and amino acid synthesis) and suppression of energy and degradative metabolism (nucleotide, tetrapyrrole, and cofactor/vitamin synthesis) (Tables S19 to S22). Suppression of tetrapyrrole and cobalamin metabolism suggests limited oxidative capacity and impaired cofactor availability (80, 81), consistent with dysbiotic state, resource-conserving phenotype under chronic microplastic stress.

Degradative pathways, including carbohydrate, nucleotide, secondary metabolite, hormone/signal, aromatic compound, and chlorine compound degradation, were broadly suppressed, supporting a shift toward simplified energy metabolism (Table S20). Glycolysis increased under PE exposure, while the TCA cycle, PPP, CO2 fixation, aerobic and anaerobic respiration, and chemoautotrophy were higher in control. This shift toward glycolytic compensation mirrors microbial strategy to reconfigure their carbon flux to mitigate toxic effects of xenobiotic exposure and maintain ATP production (82, 83). Accumulation of glycolytic intermediates in bacteria can also activate stress and virulence-associated pathways, consistent with our previous work demonstrating that a PE fiber amplifies pathogen-driven dysbiosis in *Salmonella*-inoculated cecal mesocosms (49). This mechanism is particularly important for foodborne pathogens such as *Salmonella* and *Campylobacter* associated with poultry production (84), indicating that chronic exposure to environmental contaminants, such as microplastics, can promote a gut ecosystem conducive to pathogen proliferation and virulence.

Enrichment in acetylation/inactivation and macromolecule-modification pathways further supports a stress-response phenotype. Increased envelope remodeling indicates microbial investment in membrane stabilization to counteract hydrophobic microplastic interactions (85). Post-translational modulation of proteins, including acetylation, can aid in rapid adaptation without energy-intensive protein synthesis (86). Such patterns parallel observations from microplastic-colonizing communities, where suppressed oxidative metabolism, enhanced envelope remodeling, and extensive post-translational modification accompany biofilm formation and long-term persistence on polymer surfaces. BioCyc mapping revealed that taxa enriched under PE exposure activated nucleotide salvage, carbohydrate breakdown, and envelope-precursor biosynthesis, suggesting structural maintenance rather than growth. Limited upregulation of tRNA charging and cofactor salvage pathways in *Coriobacteriaceae* and *Fournierella* suggests a response aimed at maintaining translational efficiency under PE-altered conditions. In contrast, taxa depleted under microplastic exposure, such as *Synergistes*, showed reduced respiratory and cofactor-dependent pathways.

As facultative anaerobes capable of switching between oxygen-dependent and anaerobic electron-accepting pathways (87, 88), *Synergistes* depend on stable redox gradients, and their decline indicates PE-induced disruption of cecal redox balance and removal of the niche that supports growth of respiratory generalists. Similar effects were reported in other models, where MPs disturbed redox homeostasis and glycolytic pathways (89). Collectively, these enriched and depleted taxa displayed complementary characteristics of polymer-associated biofilm adaptation. PE fiber favors microorganisms capable of low-energy persistence, structural maintenance, and salvage-oriented metabolism, while disadvantaging redox specialists dependent on oxidative pathways. Such properties allow microorganisms to maintain structural integrity and stress tolerance rather than investing in growth or respiration, suggesting that PE fiber exposure may promote surface-adapted, persistence-oriented microbial phenotypes even in the absence of significant taxonomic shifts. This metabolic simplification and glycolytic shift are characteristic of a silent dysbiosis phenotype, where functional capacity is impaired before major taxonomic or pathological changes are detectable (69).

### Cecal microbial taxa exhibit hierarchies of sensitivity and metabolic plasticity under contaminant stress

**Fig 11:**
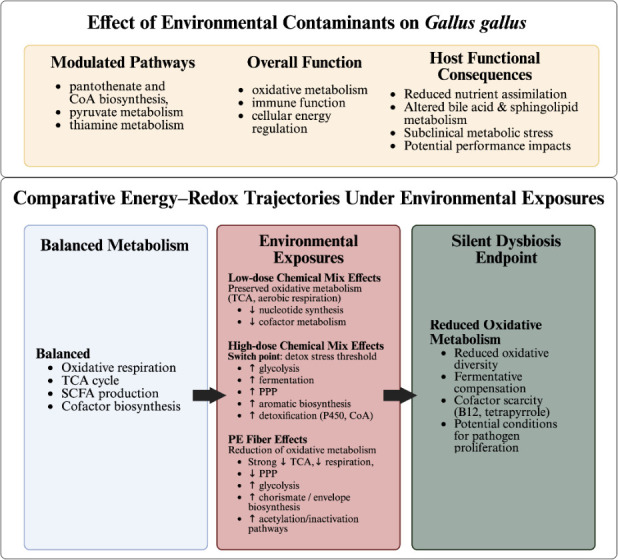
Conceptual framework illustrating how environmental contaminants reshape microbial energy metabolism in *Gallus gallus*, transitioning the gut ecosystem from a balanced oxidative state toward a silent dysbiosis endpoint with functional host consequences. Under baseline conditions (left), cecal communities maintain oxidative respiration, TCA cycle activity, SCFA production, and cofactor biosynthesis. Environmental exposures (center) drive dose-dependent metabolic divergence. Low-dose chemical mixtures cause modest reductions in nucleotide and cofactor metabolism while largely preserving oxidative pathways. High-dose mixtures cross a detoxification threshold, triggering a metabolic switch marked by elevated glycolysis, fermentation, pentose phosphate pathway activity, aromatic biosynthesis, and detoxification processes (e.g., P450, CoA). Polyethylene fibers uniquely suppress oxidative metabolism by reducing TCA activity, respiration, and PPP flux, while increasing glycolysis, chorismate/envelope biosynthesis, and acetylation–inactivation pathways. These microbial shifts converge on a silent dysbiosis endpoint (right), characterized by reduced oxidative diversity, fermentative compensation, and scarcity of key cofactors (vitamin B12, tetrapyrrole), creating conditions that may favor pathogen proliferation. Host-level functional outcomes include reduced nutrient assimilation, altered bile acid and sphingolipid metabolism, subclinical metabolic stress, and potential impacts on performance.

Across both contaminant types, exposure did not simply suppress microbial function, but restructured energy and redox metabolism via two distinct trajectories. Figure 11 summarizes how both contaminants redirect energy metabolism toward a silent dysbiosis state.

Chemical mixtures produced a dose-dependent response; the low-dose exposure preserved oxidative metabolism, including TCA activity, hydrogen production, and limited aerobic respiration, indicating that respiratory metabolism remained dominant until a critical chemical threshold was reached. High-dose exposure crossed this threshold, triggering a compensatory shift toward glycolysis, fermentation, CO2 fixation, and PPP, suggesting redirection of carbon and electron flow to maintain redox balance and support detoxification. This pattern is consistent with an energy-redox compensation phenotype, in which communities retain function by rerouting communal metabolism under xenobiotic pressure (89). PE fiber exposure produced a contrasting response. Despite modest increases in total pathway intensity, PE-exposed cecal microbiome showed broad suppression of oxidative and degradative ability, while glycolysis was the dominant energy route. Reduction in respiration, PPP, TCA cycle, CO2 fixation, and oxidative cofactor biosynthesis suggests a change toward a simplified, maintenance-oriented metabolism. Enrichment in chorismate metabolism and envelope-precursor biosynthesis further suggests a structural survival strategy associated with polymer-associated biofilm phenotypes where aromatic biosynthesis, cell-surface remodeling, and macromolecule modification support long-term persistence on hydrophobic surfaces.

Importantly, these metabolites are not restricted to microorganisms. In chemical mixture groups, enrichment of L-tryptophan degradation XI, indole derivatives, and tetrapyrrole biosynthesis points to early host-microbial redox interaction (90–92). In contrast, PE exposure reduced tetrapyrrole and cobalamin-linked pathways, implying reduced microbial contribution to vitamin and cofactor synthesis, potentially limiting SCFA production and impairing epithelial redox balance (80, 81). Ultimately, although by different routes the contaminants converged on a similar metabolic trajectory, a shift from diverse oxidative pathways toward fermentative, salvage-dependent survival. Chemical mixtures drove redox-intensive detoxification, while reduced respiratory capacity and favored low-energy persistence. Overall, contaminants reshape gastrointestinal energetics, reducing metabolic diversity and potentially creating conditions that impair nutrient cycling, favor pathogen proliferation, and promote metabolic disease.

### Implications for early biomarkers, animal health, and environmental risk assessment

Despite substantial metabolic restructuring, the broilers in the current study did not exhibit epithelial pathology or weight differences relative to control. This finding aligns with the emerging concept of the silent dysbiosis, in which compensatory metabolic shifts preserve host physiology in the short term. However, evidence from rodent and human studies showed that chronic suppression of oxidative metabolism, bile acid signaling, and cofactor biosynthesis can contribute to metabolic disorders, suppress immune function, and alter xenobiotic clearance over longer exposure periods (93–97). Since poultry are short-lived production animals, early functional disturbances are the most relevant signals of performance and food safety risk.

The metabolic simplification observed in the current study has several agricultural implications. Alterations to oxidative energy and vitamin-associated metabolism may reduce nutrient assimilation and feed efficiency, particularly under chronic high-dose exposure to agricultural chemicals. Additionally, suppression of redox specialists such as *Synergistes* and *Desulfovibrio* and restructuring to glycolytic compensation may shift the system toward a low diversity fermentative community that is more conducive to pathogen proliferation and less effective at xenobiotic detoxification. Altered bile acid and sphingolipid metabolism suggest potential for consequences associated with hepatic function, lipid homeostasis, and epithelial integrity that can be especially relevant for longer-lived birds such as layer hens and breeding stock (98, 99). Environmental risk assessments should incorporate microbiome-metabolomic end points, such as glycolytic compensatory markers, cofactor (vitamin B12/tetrapyrrole), and indicator taxa such as *Synergistes*. Including these markers can help detect subclinical changes that may affect the productivity of the host and the risk for pathogen transmission.

### Conclusion

Ultimately, the results of the current study indicate that both emerging (PE fiber) and known (ternary mixtures) agricultural groundwater contaminants induce functional remodeling rather than structural intestinal damage, altering metabolic potential without causing damage to the host. Such changes underscore the importance of microbiome and metabolome endpoints in environmental risk assessment, as they capture early physiological disruptions that would be missed by conventional histopathology alone. PE fiber did not reduce microbial diversity; however, a minor shift in community composition accompanied by a suppression of oxidative metabolism suggests a silent dysbiosis phenotype. Metabolomic profiles showed reduced cofactor biosynthesis and respiratory capacity, dysregulated bile acid and sphingolipid metabolism, and increased glycolytic compensation. Similarly, the ternary chemical mixture reshaped cecal microbial composition and induced dose-dependent metabolomic reprogramming. While oxidative metabolism was preserved under low exposure, in high-dose groups, metabolic adaptation transitioned to fermentation and detoxification-related pathways. Importantly, the real-world groundwater contaminant ranges used in the current study altered microbial community structure and function. Evaluation of combined contaminants rather than a single compound may be more relevant to real-world occurrences.

Overall, the findings suggest that agricultural and modern contaminants such as MPs can alter gut microbial energetics at a subclinical level. Chronic exposure to chemical and physical contaminants can therefore influence feed efficiency, nutrient cycling, xenobiotic clearance, and pathogen dynamics. These early metabolic perturbations may precede observable effects in longer-lived birds under chronic farm exposure. Microbiome and metabolome endpoints in agricultural safety should be incorporated into the assessment.

## Data Availability

Sequencing data were uploaded to NCBI’s Sequence Read Archive under BioProject ID PRJNA1200004. Untargeted metabolomics data were uploaded to MetaboLights under project MTBLS11958.

## SUPPLEMENTAL MATERIAL

File S1 aem.02469-25-s0001.docx Figures S1 to S6 and Tables S1 to S7.

File S2 aem.02469-25-s0002.xlsx Tables S8 to S14.

File S3 aem.02469-25-s0003.xlsx Tables S15 to S23.

ASM does not own the copyrights to Supplemental Material that may be linked to, or accessed through, an article. The authors have granted ASM a non-exclusive, world-wide license to publish the Supplemental Material files. Please contact the corresponding author directly for reuse.
