# Supplementary material for: Exposure to known and emerging groundwater contaminants significantly alters poultry microbiome and metabolome
Source: Appl Environ Microbiol. 2026 Mar 20;92(4):e02469-25. doi: 10.1128/aem.02469-25 (PMC13101470; doi:10.1128/aem.02469-25)
Supplement: File S1 — Figures S1 to S6 and Tables S1 to S7. [file aem.02469-25-s0001.docx]

Exposure to known and emerging groundwater contaminants significantly alters poultry microbiome and metabolome

**Supplemental Figures and Tables**


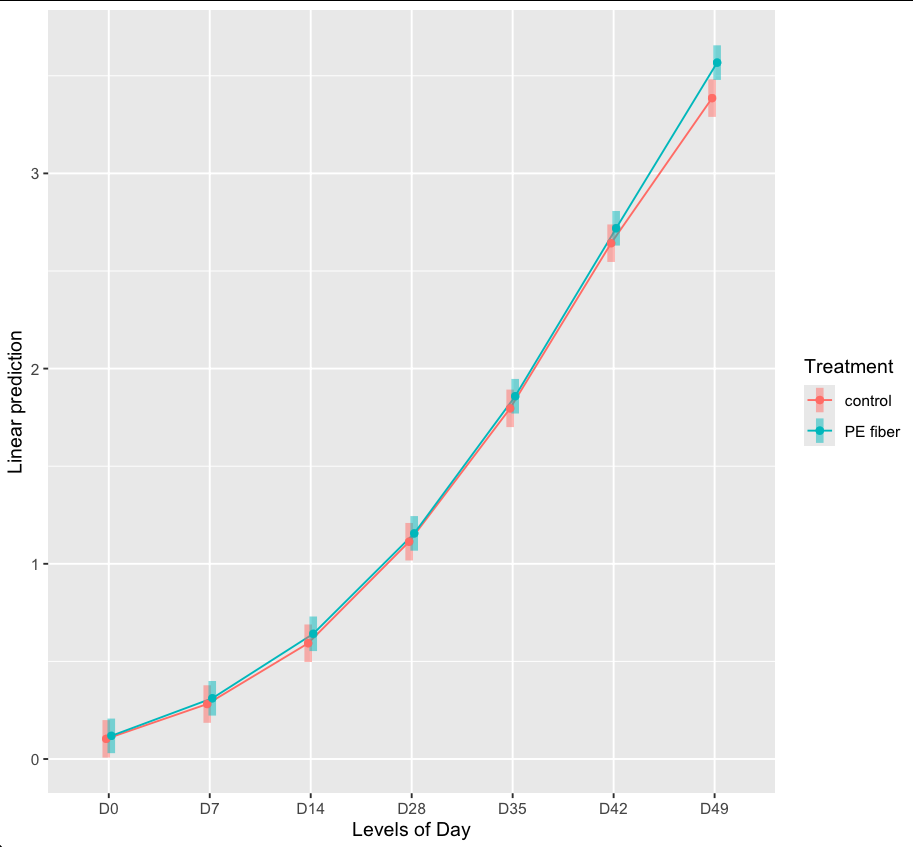


**Figure S1.** Linear Mixed Effect Model analysis assessing feed intake for control and +PE Fiber treatment groups (P ≤ 0.05).


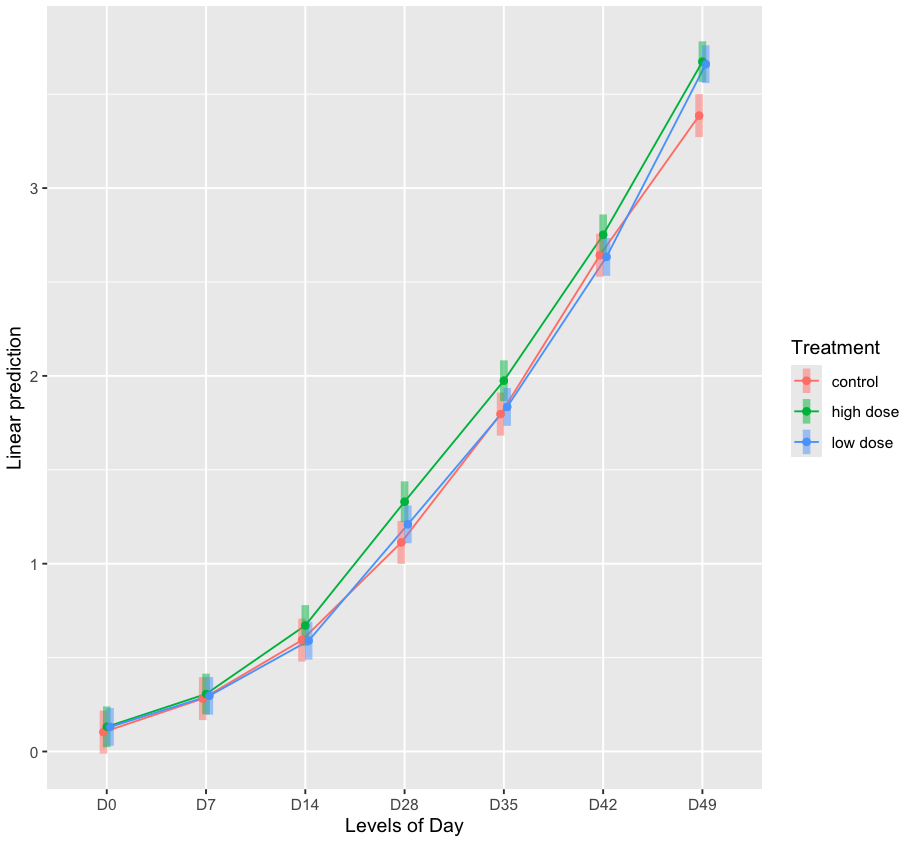


**Figure S2**. Linear Mixed Effect Model analysis assessing feed intake for control, low dose, and high dose treatment groups (P ≤ 0.05).


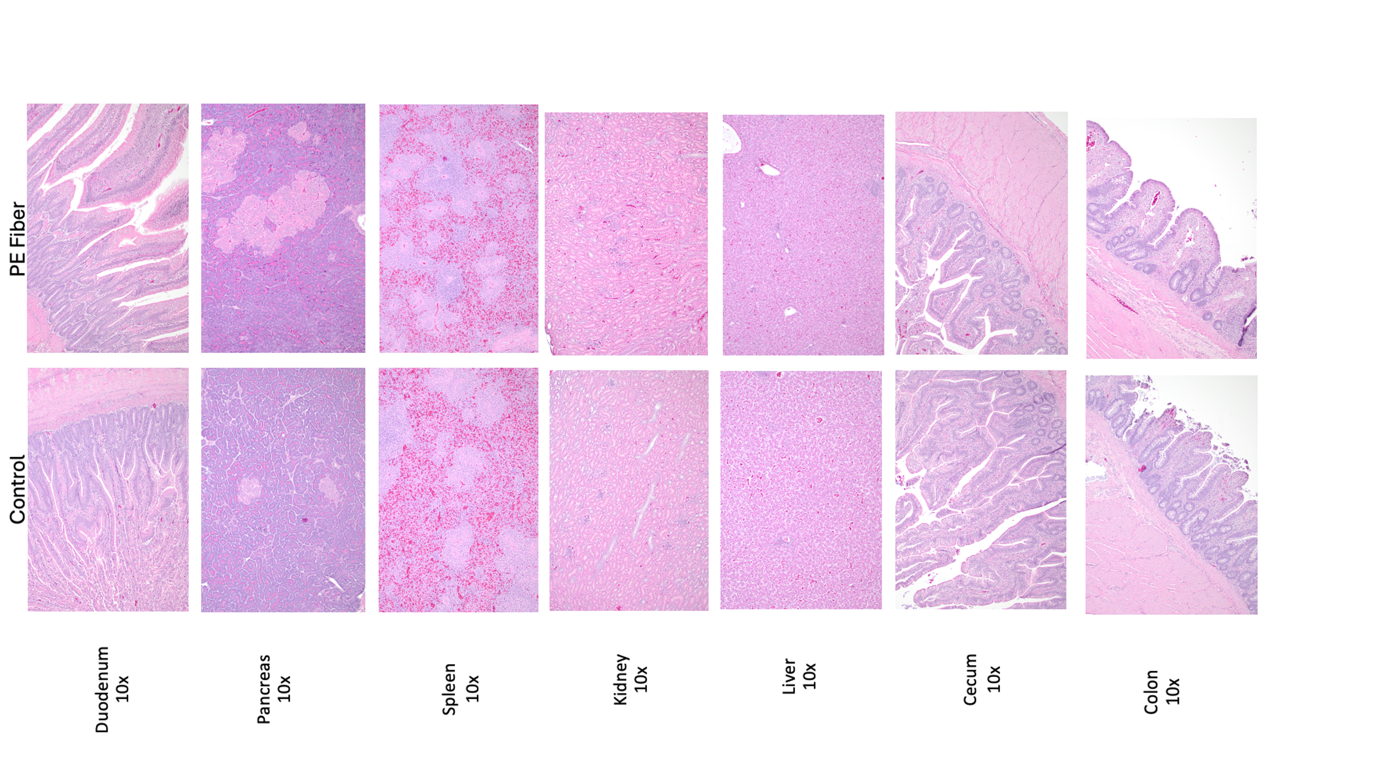


**Figure S3.** Hematoxylin and eosin staining of control and +PE fiber broilers. Sections included duodenum, pancreas, kidney, spleen, cecum, liver and colon. The images are representative of each treatment group.


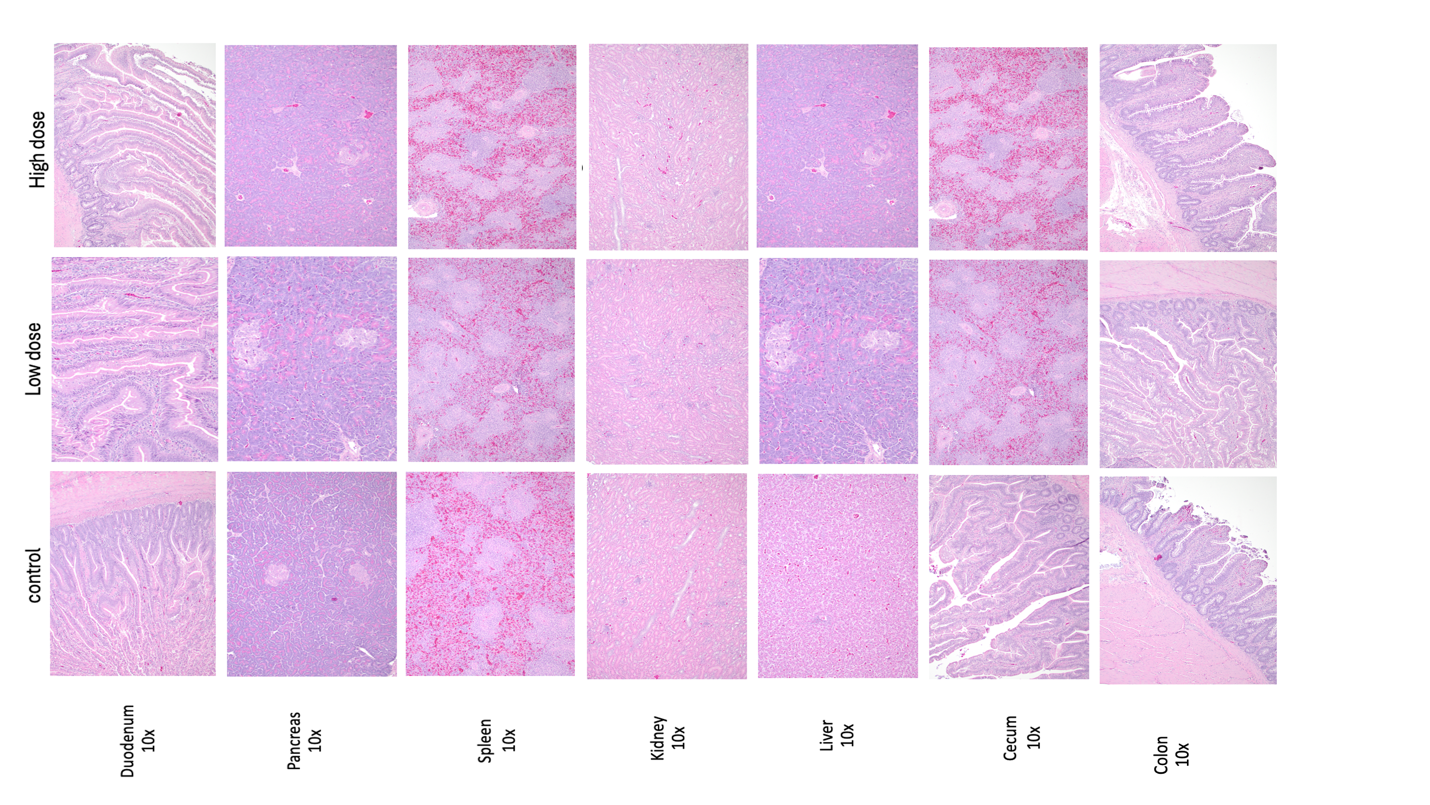


**Figure S4** Hematoxylin and eosin staining of control. Low dose and high dose broilers. Sections included duodenum, pancreas, spleen, kidney, spleen, cecum, liver and colon. The images are representative of each treatment group.


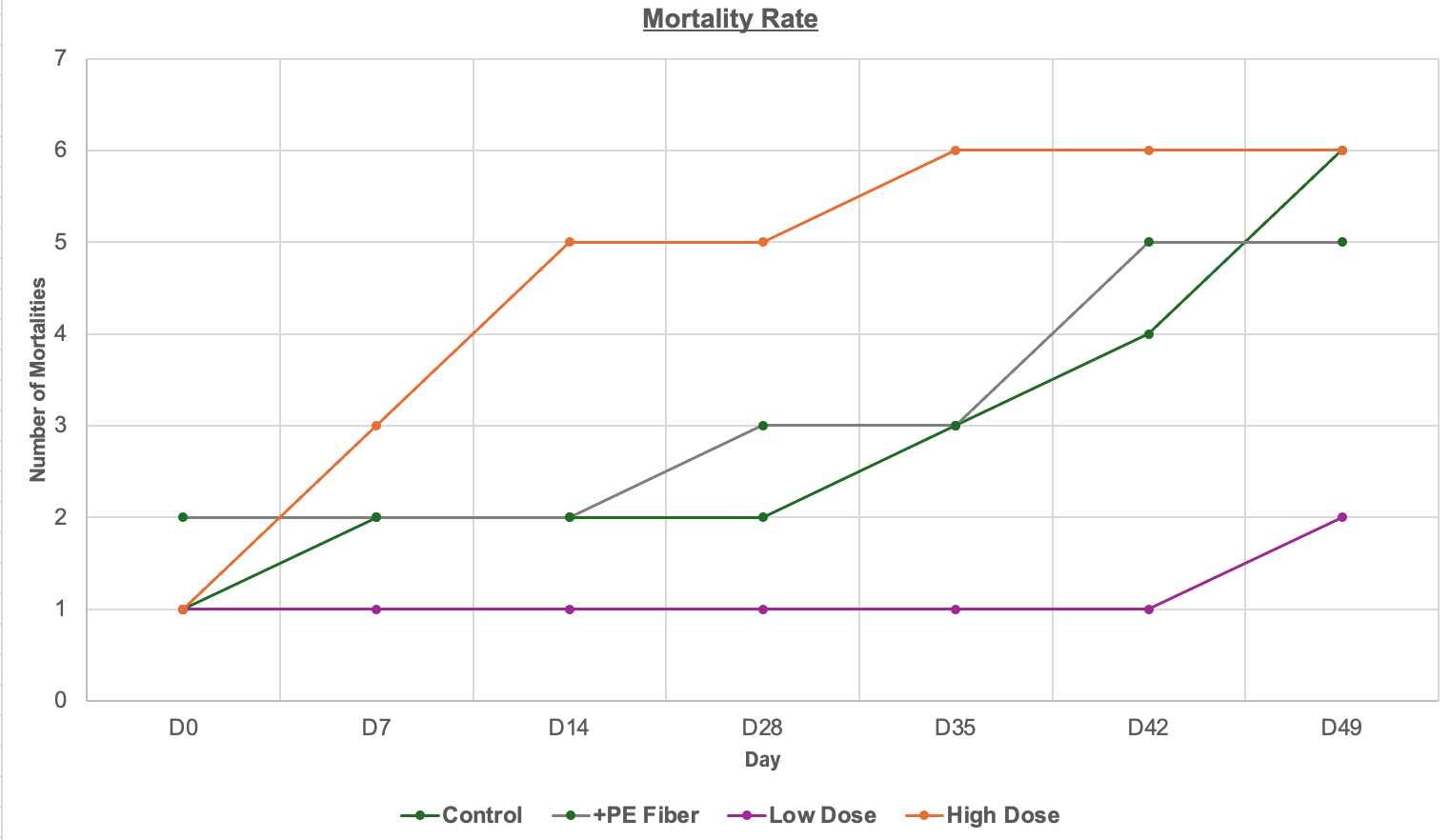


**Figure S5**. Mortality rate from the acclimation period (D0) through study termination (D49) for control (green), +PE Fiber(grey), low dose (purple) and high dose(orange).


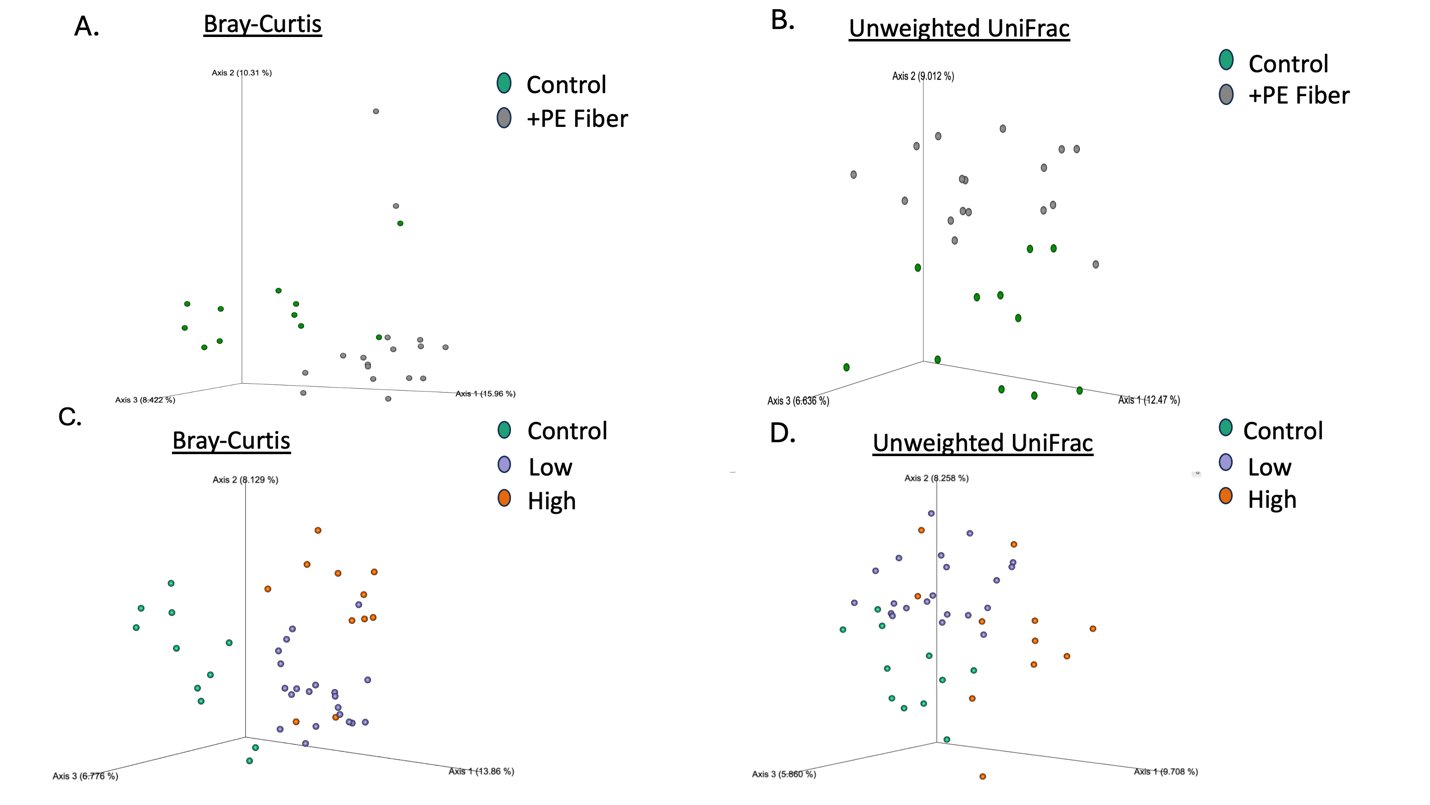


**Figure S6**. Principal component analysis for A) Bray-Curtis analysis comparing control and +PE Fiber treatment groups, B) Weighted UniFrac analysis control and +PE Fiber treatment groups, C) Bray-Curtis analysis comparing control, low dose and high dose treatment groups, and D) Weighted UniFrac analysis control, low dose and high dose treatment groups.

| Table S1. Guaranteed analysis of poultry feed.^1^ | |
| --- | --- |
| Ingredients | % |
| Crude protein | 21 |
| Lysine, minimum | 1.0 |
| Methionine, minimum | 0.42 |
| Crude Fat, minimum | 4.0 |
| Crude Fiber, maximum | 6.0 |
| Calcium, minimum | 1.0 |
| Calcium, maximum | 1.2 |
| Phosphorus, minimum | 0.55 |
| Salt, minimum | 0.15 |
| Salt, maximum | 0.5 |
| Sodium, minimum | 0.15 |
| Sodium, maximum | 0.3 |
| ^1.Agrimaster 21% Meat Producer Poultry Feed Crumbles; Blain # 334839, Mfr # 3931^ | |

| Table S2. Ingredients list of poultry feed.^1,2^ |
| --- |
| Ingredients |
| Processed Grain By-Products  Grain Products Dehulled Soybean Meal  Canola Meal  Animal Protein Products  Calcium Carbonate  Salt  DL-Methionine  Yeast Culture  L-Lysine  Propionic Acid (a preservative)  Dried Aspergillus niger Fermentation Extract  Vitamin A Supplement  Vitamin D3 Supplement  Vitamin E Supplement  Vitamin B12 Supplement  Niacin Supplement  d-Calcium Pantothenate  Folic Acid  Menadione Sodium Bisulfite Complex (source of Vitamin K activity)  Riboflavin Supplement  Pyridoxine Hydrochloride  Thiamine Mononitrate  Biotin  Manganous Oxide  Manganese Sulfate  Ferrous Sulfate  Copper Chloride  Copper Sulfate  Zinc Oxide  Zinc Sulfate  Ethylenediamine Dihydroiodide  Sodium Selenite  Dried Bifidobacterium thermophilum Fermentation Product  Dried Enterococcus faecium Fermentation Product  Dried Lactobacillus acidophilus Fermentation Product  Dried Lactobacillus casei Fermentation Product. |
| 1. ^Agrimaster 21% Meat Producer Poultry Feed Crumbles; Blain # 334839, Mfr # 3931^ 2. ^Percentages not available for ingredients list.^ |

| Table S3. Analysis of control (n=11), low dose (n=21) and high dose (n=11) treatment effects based on α-diversity metrics as determined with ANOVA. | | | | | |
| --- | --- | --- | --- | --- | --- |
| Metric | Model | Sum_sq | Df | F | PR(>F) |
| Faith’s Phylogenetic Diversity | Treatment | 43.79 | 2.0 | 10.48 | 0.00 |
|  | Residual | 83.57 | 40.0 |  |  |
| Shannon’s entropy | Treatment | 0.84 | 2.0 | 3.48 | 0.04 |
|  | Residual | 4.84 | 40.0 |  |  |
| Pielou’s evenness | Treatment | 0.00 | 2.0 | 1.32 | 0.29 |
|  | Residual | 0.04 | 40.0 |  |  |
| Observed Features | Treatment | 21,140.86 | 2.0 | 7.75 | 0.00 |
|  | Residual | 54,524.21 | 40.0 |  |  |

| Table S4. Kruskal-Wallis pairwise analysis results for a-diversity metrics. | | | | |
| --- | --- | --- | --- | --- |
| **Group 1** | **Group 2** | **a -diversity metrics ^a^** | | |
|  |  | Shannon’s Diversity Index | | |
|  |  | H | P-value | Q-value |
| High dose mixture  (n =11) | Control  (n= 11) | 2.59 | 0.11 | 0.16 |
| Low dose mixture  (n =21) | Control  (n= 11) | 0.66 | 0.41 | 0.41 |
| High dose mixture  (n =11) | Low dose mixture  (n= 21) | 6.14 | **0.01** | **0.04** |
|  |  | Pielou’s Evenness | | |
|  |  | H | P-Value | Q-Value |
| High dose mixture  (n =11) | Control  (n= 11) | 1.99 | 0.16 | 0.41 |
| Low dose mixture  (n =21) | Control  (n= 11) | 0.33 | 0.56 | 0.56 |
| High dose mixture  (n =11) | Low dose mixture  (n= 21) | 1.19 | 0.26 | 0.41 |
|  |  | Faith’s phylogenetic Diversity | | |
|  |  | H | P-Value | Q-Value |
| High dose mixture  (n =11) | Control  (n= 11) | 7.07 | **0.01** | **0.01** |
| Low dose mixture  (n =21) | Control  (n= 11) | 0.01 | 0.92 | 0.92 |
| High dose mixture  (n =11) | Low dose mixture  (n= 21) | 11.78 | **0.00** | **0.00** |
|  |  | Observed Features | | |
|  |  | H | P-Value | Q-Value |
| High dose mixture  (n =11) | Control  (n= 11) | 7.25 | **0.01** | **0.01** |
| Low dose mixture  (n =21) | Control  (n= 11) | 0.01 | 0.94 | 0.94 |
| High dose mixture  (n =11) | Low dose mixture  (n= 21) | 7.61 | **0.01** | **0.01** |
| ^a.^ Bolded values indicate significance (P-value ≤ 0.05; Q-value ≤ 0.05). | | | | |

| Table S5. Analysis of control (n=11), low dose (n=21) and high dose (n=11) treatment effects based on β-diversity metrics as determined with PERMANOVA (P-value ≤ 0.05).^a^ | | | | |
| --- | --- | --- | --- | --- |
| Metric | Test statistic | Sample size | Test statistic | P-value |
| Bray-Curtis | Pseudo-F | 43 | 4.09 | 0.00 |
| Jaccard | Pseudo-F | 43 | 2.31 | 0.00 |
| Weighted UniFrac | Pseudo-F | 43 | 3.84 | 0.00 |
| Unweighted UniFrac | Pseudo-F | 43 | 2.46 | 0.00 |
| ^a.^ PERMANOVA performed with 999 permutations. | | | | |

| Table S6. Kruskal-Wallis pairwise analysis results for a-diversity metrics comparing control and +PE Fiber treatment groups. | | | | |
| --- | --- | --- | --- | --- |
| **Group 1** | **Group 2** | **a-diversity metric** | | |
|  |  | Shannon’s Diversity Index | | |
| +PE Fiber  (n=17) | Control  (n=11) | H | P-Value | Q-Value |
|  |  | 2.95 | 0.09 | 0.09 |
|  |  | Pielou’s Evenness | | |
| +PE Fiber  (n=17) | Control  (n =11) | H | P-Value | Q-Value |
|  |  |  |  |  |
|  |  | 0.93 | 0.33 | 0.33 |
|  |  | Faith’s phylogenetic Diversity | | |
| +PE Fiber  (n=17) | Control  (n=11) | H | P-Value | Q-Value |
|  |  | 2.63 | 0.1 | 0.1 |
|  |  | Observed Features | | |
| +PE Fiber  (n=17) | Control  (n=11) | H | P-Value | Q-Value |
|  |  | 2.2 | 0.14 | 0.14 |

| Table S7. Analysis of control (n=11) and +PE Fiber (n=17) treatment effects based on β-diversity metrics as determined with PERMANOVA (P-value ≤ 0.05; Q-value ≤ 0.05).^a^ | | | | | |
| --- | --- | --- | --- | --- | --- |
| Metric | Test statistic | Sample size | Test statistic | P-value | Q-value |
| Bray-Curtis | Pseudo-F | 28 | 4.09 | 0.00 | 0.00 |
| Jaccard | Pseudo-F | 28 | 2.12 | 0.00 | 0.00 |
| Weighted UniFrac | Pseudo-F | 28 | 2.21 | 0.00 | 0.00 |
| Unweighted UniFrac | Pseudo-F | 28 | 2.16 | 0.00 | 0.00 |
| ^a.^ PERMANOVA performed with 999 permutations. | | | | | |
